# Suspension Electrolytes with Catalytically Self‐Expediating Desolvation Kinetics for Low‐Temperature Zinc Metal Batteries

**DOI:** 10.1002/adma.202501079

**Published:** 2025-03-23

**Authors:** Jing Dong, Xiaomin Cheng, Haifeng Yang, Huihua Li, Haitao Liu, Lujie Jia, Yongzheng Zhang, Qinghua Guan, Jiqiang Jia, Fanglin Wu, Jing Zhang, Meinan Liu, Hongzhen Lin, Jian Wang

**Affiliations:** ^1^ School of Nano‐Tech and Nano‐Bionics University of Science and Technology of China Hefei 230026 China; ^2^ *i*‐Lab & CAS Key Laboratory of Nanophotonic Materials and Devices Suzhou Institute of Nano‐Tech and Nano‐Bionics Chinese Academy of Sciences Suzhou 215123 China; ^3^ Helmholtz Institute Ulm (HIU) D89081 Ulm Germany; ^4^ Karlsruhe Institute of Technology (KIT) D76021 Karlsruhe Germany; ^5^ Laboratory of Computational Physics Institute of Applied Physics and Computational Mathematics Beijing 100088 China; ^6^ State Key Laboratory of Chemical Engineering East China University of Science and Technology Shanghai 200237 China; ^7^ Advanced Materials Analysis and Test Center School of Materials Science and Engineering Xi'an University of Technology Xi'an 710048 China

**Keywords:** inner helmholtz plane, localized electric field, rapid desolvation, suspension electrolyte engineering, Zn metal battery

## Abstract

The conventional electrolyte for rechargeable aqueous zinc metal batteries (AZMBs) breeds many problems such as Zn dendrite growth and side reaction of hydrogen evolution reaction, which are fundamentally attributed to the uneven ion flux owing to the high barriers of desolvation and diffusion of Zn[(H_2_O)_6_]^2+^ clusters. Herein, to modulate the [Zn(H_2_O)_6_]^2+^ solvation structure, the suspension electrolyte engineering employed with electron‐delocalized catalytic nanoparticles is initially proposed to expedite desolvation kinetics. As a proof, the electron‐density‐adjustable CeO_2‐_ *_x_* is introduced into the commercial electrolyte and preferentially adsorbed on the Zn surface, regulating the Zn[(H_2_O)_6_]^2+^ structure. Meanwhile, the defect‐rich CeO_2‐_ *_x_* redistributes the localized space electric field to uniformize ion flux kinetics and inhibits dendrite growth, as confirmed by a series of theoretical simulations, spectroscopical and experimental measurements. Encouragingly, the CeO_2‐_ *_x_* decorated suspension electrolyte enables a long stability over 1200 cycles at 5 mA cm^−2^ and an extended lifespan exceeding 6500 h with lower overpotentials of 34 mV under 0 °C. Matched with polyaniline cathodes, the full cells with suspension electrolyte exhibit a capacity‐retention of 96.75% at 1 A g^−1^ under −20 °C as well as a long lifespan of up to 400 cycles in a large‐areal pouch cell, showcasing promising potentials of suspension electrolyte for practical AZMBs.

## Graphical abstract

The CeO_2‐_ *_x_*‐suspension electrolyte is initially proposed to regulate the Zn[(H_2_O)_6_]^2+^ solvation structure and alters the inner Helmholtz plane to accelerate Zn[(H_2_O)_6_]^2+^ desolvation, achieving a homogenized electric field and uniform ion flux kinetics. Consequently, the suspen sion electrolyte achieves a service life exceeding 6500 h at 0 °C and demonstrates outstanding rate performance at −20 °C, highlighting its practical potential for AZMBs.

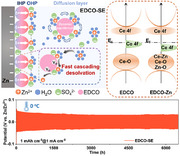

## 1 Introduction

**Figure 1:**
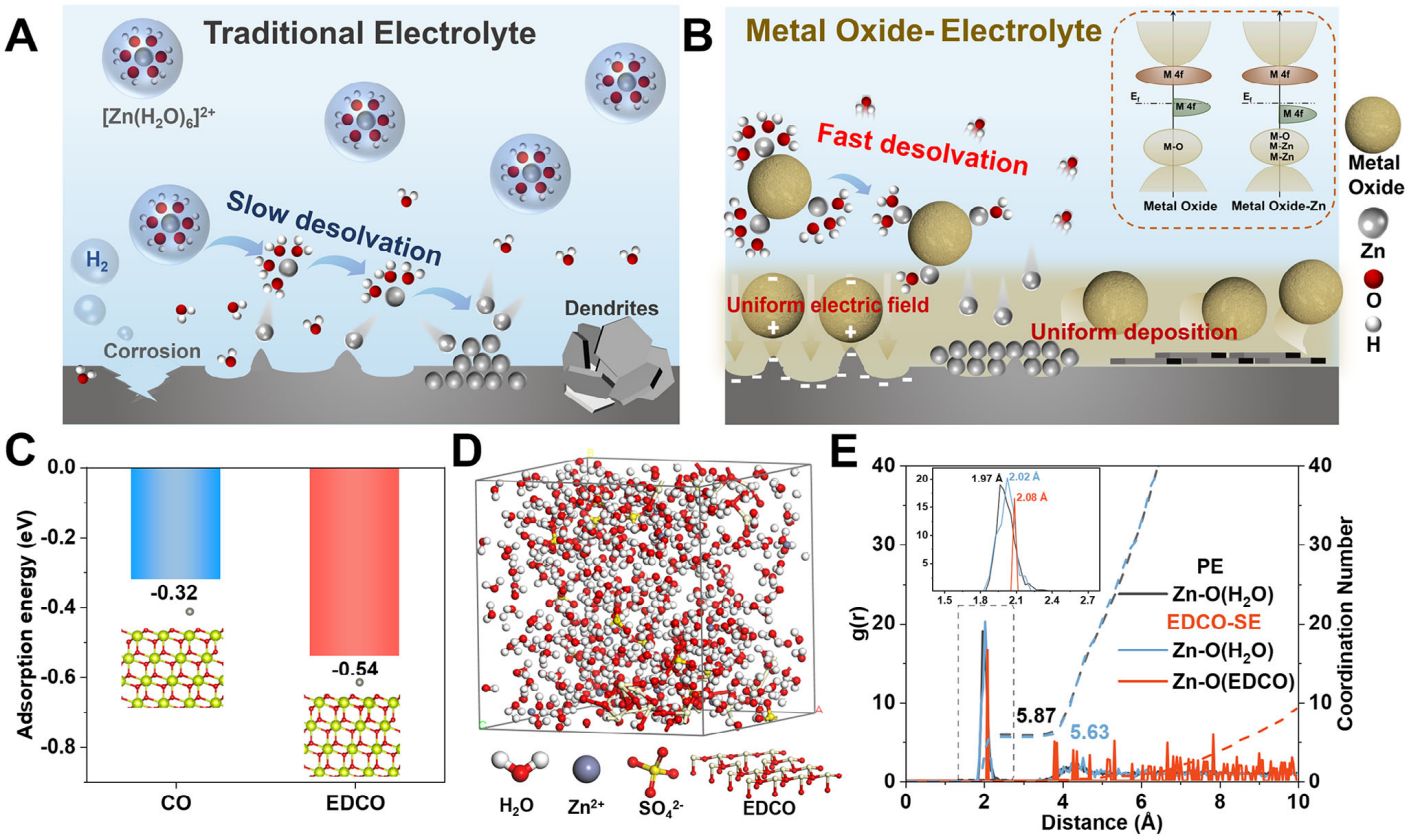
Schematic illustration and calculation of EDCO effect on electrolyte. Schematic illustration of Zn^2+^ solvation structure and Zn deposition behavior in A) traditional electrolyte and B) metal oxide‐traditional electrolyte. C) Adsorption energy of Zn on CO and EDCO. D) The 3D snapshot of the EDCO‐SE obtained from MD simulations. E) RDFs for Zn‐O(EDCO) and Zn‐O(H_2_O) in the EDCO‐SE.

Rechargeable aqueous zinc metal batteries (AZMBs) represent a promising technology for future large grid storage systems.^[^ ^1^ ^]^ In comparison to other anodic electrodes, the metallic Zn anode attracts significant attention due to its high theoretical capacity (5855 mAh cm^−3^ and 819 mAh g^−1^), low redox potential (−0.763 V vs. standard hydrogen electrode) and environmental friendliness.^[^ ^2^ ^]^ However, numerous challenges also block the development of AZMBs such as Zn dendrite growth and side reaction of hydrogen evolution reaction (HER), which are fundamentally attributed to the active water in the [Zn(H_2_O)_6_]^2+^ solvation shell and inhomogeneous Zn^2+^ flux (**Figure** **1**A), causing deteriorated electrochemical performance and depressive lifespan.^[^ ^3^ ^]^ Decreasing the operation temperature down to a lower temperature such as −20 °C, the original [Zn(H_2_O)_6_]^2+^ solvation shell would become larger and larger, inducing the severe desolvation/diffusion barrier and poor cycling stability with a short lifespan.^[^ ^4^ ^]^

To tackle the above‐mentioned issues, various surface modifications and phase constructions have been proposed to separate the active water molecules in the [Zn(H_2_O)_6_]^2+^ solvation shell from electron contact, inhibiting the occurrence of HER. Although this artificial interphase engineering is endowed with surface protection,^[^ ^5^ ^]^ the whole fabrication processes seem to be extremely tedious and complex owing to the existence of several steps of coating and drying, costing too much energy and contaminating the environment. Meanwhile, the additional polymeric binders and excessive layer thickness would hinder Zn^2+^ transport, slowing ion migration and limiting desolvation and diffusion kinetics. As known, the inner Helmholtz plane (IHP) is particularly important for the desolvation, diffusion, and electrochemical deposition of Zn^2+^, playing a crucial role in controlling interfacial reactions.^[^ ^6^ ^]^ Alternatively, the electrolyte optimizations are also another option for regulating the solvation structure of [Zn(H_2_O)_6_]^2+^. In the commercial pristine electrolyte (PE), the IHP is filled up with partially or previously dissociated Zn(H_2_O)*_x_* ^2+^, water dipoles, and anions.^[^ ^7^ ^]^ To regulate the IHP, the current mainstream strategy is to increase the concentration of electrolyte salt to an extremely high extent to change the Zn^2+^ solvation sheath, forming the increased ratio of contact ion pairs (CIP) or even ion aggregates (AGGs) to facilitate the desolvation kinetics.^[^ ^8^ ^]^ Indeed, the Zn ion transport is significantly increased through this crowded method. However, this approach inevitably suffers from high viscosity, slow ionic kinetics, high cost, and the potential corrosion toward Zn metal or current collectors.^[^ ^9^ ^]^ Therefore, it is necessary to figure out another feasible method to modulate the solvation shell structure.

As accepted, the desolvation of [Zn(H_2_O)_6_]^2+^ in the Helmholtz layer is the rate‐limiting step for Zn^2+^ transport and plating, but the high desolvation energy barrier leads to sluggish kinetics.^[^ ^10^ ^]^ The high diffusion energy barrier also hinders the efficient transfer of zinc ions between the outer Helmholtz plane (OHP) and IHP, resulting in uneven zinc ion flux and unexpected dendrite formation. Regulating Helmholtz layer composition, surface electric field, and desolvation processes is critical for inhibiting dendrite growth and retarding side reaction of HER.^[^ ^11^ ^]^ Representatively, electrolyte additives such as organic materials^[^ ^12^ ^]^ and metal ions,^[^ ^13^ ^]^ have been put forward, which can be attracted and adsorbed on the Zn surface to regulate the IHP. However, these electrolyte additives are not effective to facilitate the desolvation kinetics of [Zn(H_2_O)_6_]^2+^ owing to the existed higher barriers of desolvation and diffusion at the interface. Furthermore, these electrolyte additives also participate in the solid electrolyte interphase (SEI) formation,^[^ ^14^ ^]^ but it may get failed after long‐term cycling because of continuous consumption. Thus, constructing sustainable additives in the aqueous electrolyte remains highly desired.

As known, combining the electron delocalization strategy with the metal oxide nanoparticles would help to achieve numerous zincophilic sites,^[^ ^15^ ^]^ which exhibits the regulation of local space electric field for realizing uniform Zn flux.^[^ ^16^ ^]^ Certain metal oxides with unique electronic structures can alter both the solvation shell and IHP composition. Our previous studies have demonstrated that tuning the *4f*‐electrons in metal oxide catalysts via Schottky defects can modify the electronic structure, enhancing catalytic efficiency.^[^ ^17^ ^]^ Introducing the electron‐adjustable catalysts into the electrolyte would self‐expediate the interfacial desolvation kinetics as well.^[^ ^18^ ^]^ Meanwhile, the redistributed electron density helps to form more active sites for desolvation, effectively influencing the net electric field in the micro‐space. Thus, incorporating the catalytic metal oxides into the aqueous electrolyte seems to be fantastic and effective (Figure 1B), forming the new suspension electrolyte (SE), which is expected to realize fast desolvation and uniform Zn plating.

In this work, an advanced suspension electrolyte engineering by employing electron‐delocalized CeO_2‐_ *_x_* (EDCO) catalytic nanoparticles with the aqueous commercial pristine electrolyte is proposed to modulate the behaviors of Zn^2+^ and active water molecules at the IHP, serving as kinetic‐improved suspension electrolyte with decreased barriers. Theoretical simulations and experimental measurements reveal that catalytic EDCO not only regulates the [Zn(H_2_O_6_])^2+^ solvation structure but also expedites the desolvation process through Schottky defect‐derived catalytic sites, exhibiting a partial portion of EDCO is dynamically adsorbed onto the Zn anode surface. Moreover, the electron‐adjustable CeO_2‐_ *_x_* initially and effectively redistributes the local electric field, achieving a homogenized electric field and uniform ion flux kinetics, as confirmed by various simulations, in situ sum frequency generation (SFG) spectroscopy, time‐of‐flight secondary ion mass spectrometry (TOF‐SIMS) and optical image evolutions. Encouragingly, the side reaction of HER caused by active water from [Zn(H_2_O)_6_]^2+^ solvation has been prevented and the corresponding corrosion is also inhibited. As a proof‐of‐concept, the Zn anode with EDCO‐decorated suspension electrolyte (EDCO‐SE) shows a higher Zn^2+^ transference number (0.70), achieves a high Coulombic efficiency (CE) of 99.92% within 1200 cycles at 5 mA cm^−2^ and long‐term lifespan of more than 6500 h with lower overpotentials of 34 mV even under 0 °C. Matched with polyaniline (PANI) cathodes, full cells with EDCO‐SE exhibit enhanced cycle life and capacity retention of 89.23% after 3500 cycles at 2 A g^−1^. Reduced the surrounding temperature to −20 °C, the full cell exhibits a capacity‐retention of 96.75% at 1 A g^−1^ after 2400 cycles. More impressively, the large areal pouch cell also stabilizes for 400 cycles at 1 A g^−1^, highlighting the practical potential of catalytic suspension electrolyte for AZMBs.

## 2 Results and Discussion

To elucidate the desolvation mechanism of the EDCO suspension catalyst, density functional theory (DFT) calculations were performed to analyze the adsorption energy of Zn^2+^ on CeO_2_ (CO) or EDCO surfaces, further confirming the intrinsic driving force of EDCO in accelerating desolvation.^[^ ^19^ ^]^ As shown in Figure 1C, the adsorption energy of a single Zn atom on the stoichiometric CO(111) surface was calculated as −0.32 eV. Surprisingly, the calculated adsorption energy on the EDCO(111) surface with oxygen vacancies was determined to be −0.54 eV, and the stronger adsorption between Zn and EDCO can effectively promote the desolvation of Zn^2+^. In comparison, the binding energy between Zn^2+^ and single H_2_O is also calculated and it is ≈4.9 eV, which is much higher than that of Zn on CO or EDCO (Figure S1, Supporting Information). This means CO or EDCO does not simply replace the H_2_O molecules in the [Zn(H_2_O)_6_]^2+^ like a ligand. Instead, the EDCO will adsorb the [Zn(H_2_O)_6_]^2+^ species and exert the forces on the Zn^2+^‐H_2_O bond thanks to the local field created by the defective sites. To investigate the interactions and coordination number in the electrolyte shell, molecular dynamics (MD) simulations were employed to analyze Zn^2+^ solvation behavior and the spatial distribution.^[^ ^20^ ^]^ A layer of (111) crystal face of EDCO, the most exposed under natural conditions, was selected to interact with the ZnSO_4_‐based aqueous electrolyte (Figure 1D). Figure 1E displays the radial distribution functions (RDFs) to examine the coordination structure of Zn^2+^.^[^ ^21^ ^]^ In the PE, the peak at 1.97 Å with a coordination number of 5.87 corresponds to the distance between the Zn^2+^ and the O of H_2_O (Figure S2, Supporting Information). In contrast, the EDCO‐SE exhibits two kinds of Zn‐O(H_2_O) and Zn‐O(EDCO) coordination peaks at 2.02 and 2.08 Å, corresponding to coordination numbers of 5.63 and 0.007, respectively. As long as the Zn^2+^‐H_2_O bond is cleaved, the resulting high‐energy Zn^2+^ and free H_2_O have the freedom to escape from the adsorption independently, leading to refreshment of the active site. That is why a moderate adsorption strength between the nanoparticles and the zinc ions is favored, but not the stronger the better. These findings demonstrate that EDCO redistributes the primary solvation shell of Zn^2+^, altering the solvation structure and enlarging the Zn‐O bond for fast desolvation and inhibiting the severe side reactions of hydrogen evolution reaction derived from strong Zn^2+^‐H_2_O bond.^[^ ^22^ ^]^

**Figure 2:**
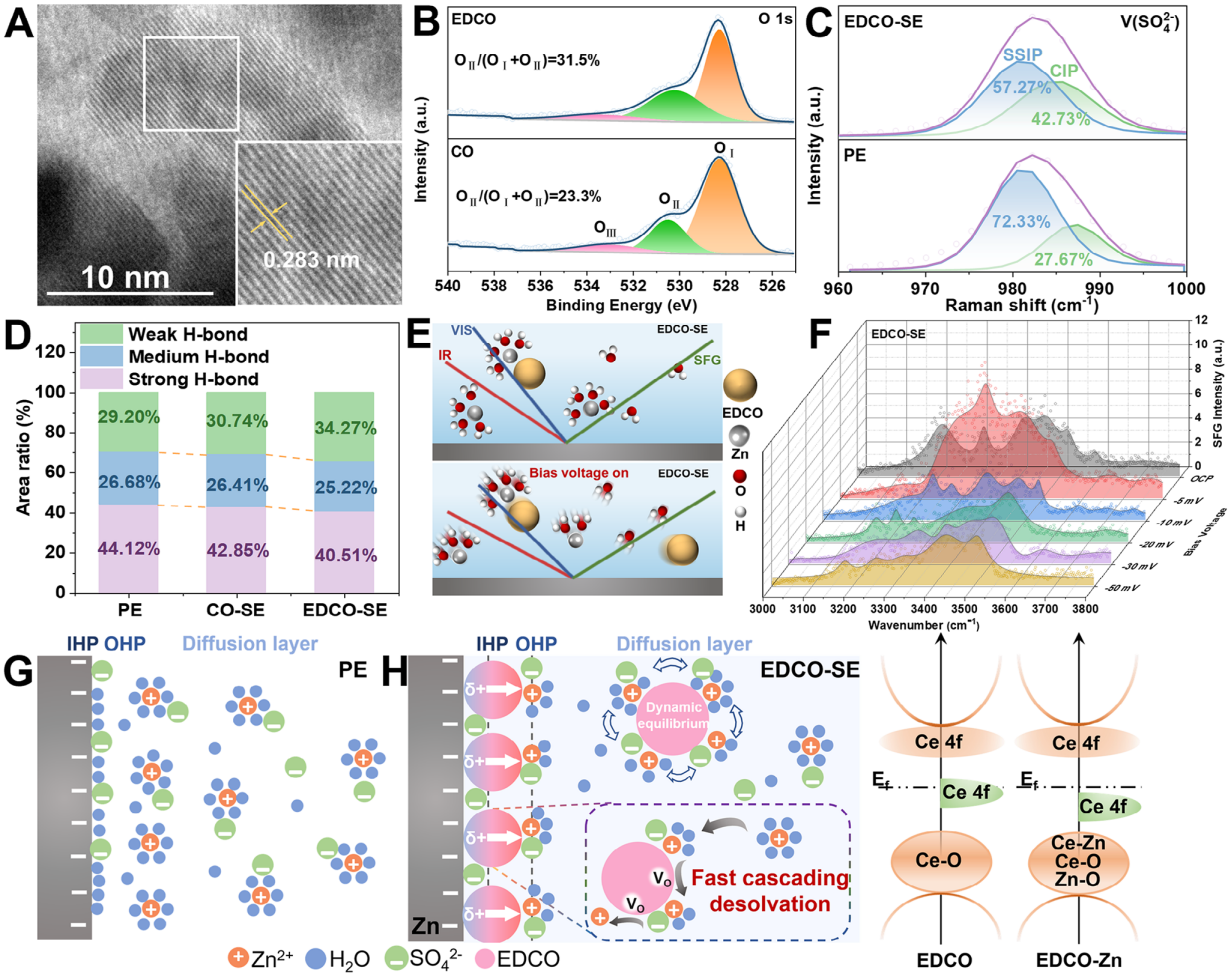
Characterizations of the EDCO and regulation of the Helmholtz plane by EDCO. A) HRTEM image of EDCO. B) The high‐resolution XPS spectra comparison of O 1s for CO and EDCO. C) Raman spectra of various electrolytes. D) Comparison of hydrogen bonding components of various electrolytes. E) Schematic illustration of Zn^2+^ dissociation with/without the bias voltage in EDCO‐SE. F) SFG‐intensity changes of O‐H region in EDCO‐SE. Schematic illustrations of the EDL structure for Zn electrode in G) PE and H) EDCO‐SE.

The electron‐delocalized CeO_2‐_ *_x_* was achieved by hydrothermal reaction and hydrogen reduction (see the details in Supporting Information). Transmission electron microscopy (TEM) reveals that the EDCO nanoparticles exhibit a uniform cubic morphology of ≈12 nm (Figure S3, Supporting Information). The high‐resolution TEM (HRTEM) image in **Figure** **2**A further confirms the high crystallinity of EDCO with an interplanar spacing of 0.283 nm compared to CO (0.279 nm) (Figure S4, Supporting Information). Besides, the size effect of nanoparticles was also investigated and the smaller size will facilitate the stability in the SE for, which can maintain the uniform dispersion for 2h. And the particle size distribution measurements show there is no obvious change and aggregation by stirring the precipitations in the SE (Figures S5 and S6, Supporting Information). The X‐ray diffraction (XRD) patterns with the Rietveld refinement analysis confirm that both CO and EDCO adopt a cubic structure (JCPDS 34–0394) after thermal treatment (Figures S7 and S8, Supporting Information). These results indicate that the thermal treatment did not induce any significant changes in the crystal structure, meaning that the lattice of CO remains intact, thus demonstrating the structural stability of both CO and EDCO. Three distinct peaks at 529.6, 531.5, and 532.5 eV, corresponding to lattice oxygen (O_I_), oxygen vacancies (O_II_), and adsorbed oxygen species (O_III_), respectively, are observed in the high‐resolution O 1s X‐ray photoelectron spectroscopy (XPS) analysis.^[^ ^23^ ^]^ The quantified ratio of oxygen vacancies, defined as O_II_ / (O_I_ + O_II_), shows a significant increase in EDCO (31.5%) from 23.3% of CO (Figure 2B). Meanwhile, Ce^3+^ and Ce^4+^ are detected and the relative proportion of Ce^3+^ increases in EDCO due to the generation of oxygen vacancies (Figure S9, Supporting Information),^[^ ^24^ ^]^ which is consistent with the results of the O 1s XPS spectra. To further evaluate the effect of Schottky defects, the Mott–Schottky plots derived from electrochemical impedance spectroscopy (EIS) were obtained (Figure S10, Supporting Information).^[^ ^17^, ^25^ ^]^ Both CO and EDCO exhibit *n*‐type semiconductor behavior, however, the EDCO displays a reduced slope, indicative of a higher donor density (N_d_).^[^ ^26^ ^]^ These results clearly demonstrate that the abundance of oxygen defects in EDCO results in electron delocalization, regulating the local electric field and enhancing its catalytic capability.^[^ ^27^ ^]^

At the beginning, the suspension particle concentration effect ranging from 1 to 10 mg mL^−1^ on the solvation shell and electrochemical performance was roughly estimated. Under the concentration of 3 mg mL^−1^, the overpotential is minimized and the cycling lifespan is the longest, thus the 3 mg mL^−1^ is adopted as the experimental concentration for EDCO (Figure S11, Supporting Information). To trace the accelerated desolvation kinetics in the EDCO‐SE, Raman and in situ SFG spectroscopies were used to elucidate the hydrated solvation structure. In the PE, the ν‐SO_4_^2−^ exhibits two ion‐pair species, the solvent‐separated ion pair (SSIP, [Zn^2+^(H_2_O)_6_·SO_4_^2−^]) and the contact ion pair (CIP, [Zn^2+^(H_2_O)_5_·OSO_3_^2−^]),^[^ ^28^ ^]^ where the relative content of CIP gradually increases with the concentration of electrolyte salts due to strong Zn^2+^‐SO_4_^2−^ coupling (Figure S12, Supporting Information). Notably, in the EDCO‐SE, the CIP percentage increases significantly to 42.73%, compared to 27.67% in the PE (Figure 2C). Meanwhile, the introduction of EDCO also alters the hydrogen bonding network, of which the weak, medium, and strong hydrogen bonds are deconvoluted (Figure 2D; Figure S13, Supporting Information).^[^ ^29^ ^]^ In the PE, the proportion of strong H‐bond is the widest peak area of 44.12%, indicating the high reactivity of free hydrogen bonds. In comparison, the proportion of strong hydrogen bonds in the CO‐suspension electrolyte (CO‐SE) decreases and in EDCO‐SE further reduces to 40.51%. This indicates that EDCO disrupts the original network and reduces the reactivity of free water molecules, alleviating the side reactions of HER.^[^ ^30^ ^]^ The molecular vibrations at the electrode/electrolyte interface were further probed by in situ interface‐sensitive SFG spectroscopy,^[^ ^31^ ^]^ as illustrated in Figure 2E. Apparent solvent peaks at ≈3200 and 3500 cm^−1^ of the O‐H bond are observed, indicating the presence of free solvents and solvated solvents at the interface (Figure 2F).^[^ ^32^ ^]^ At the open circuit potential (OCP) state, it is evident that the intensity of the O‐H band at the EDCO‐SE interface is much lower than that at the PE interface. Upon applying a bias voltage of 5 mV, the O‐H vibrational intensity of EDCO‐SE increased, possibly due to the bias voltage causing partial adsorption of water molecules on the Zn surface. When the applied bias voltage is further increased, a significant decrease in the O‐H bond peak intensity of EDCO‐SE can be observed, and the peak shape becomes more complex. This suggests that EDCO catalyzes the dissociation of [Zn(H_2_O)_6_]^2+^ for rapid generation of free Zn^2+^ and reducing the content of active water molecules.^[^ ^33^ ^]^ In stark contrast, even under an applied bias voltage of 150 mV, the peak intensity of PE remains largely unchanged, indicating that a significant amount of H_2_O and solvated [Zn(H_2_O)_*x*_]^2+^ persists on the Zn surface (Figure S14, Supporting Information). As illustrated in Figure 2G,H, the electrical double layer (EDL) composition for the Zn electrode surface was reconstructed with the addition of EDCO catalytic particles. In the diffusion layer of EDCO‐SE, due to its catalytic effect on desolvation and adsorption of Zn^2+^ by EDCO, the EDCO holds multiple Zn^2+^ with varying numbers of coordinated water molecules, achieving a dynamic equilibrium. When EDCO particles approach the Zn electrode surface, Zn^2+^ undergoes rapid desolvation for future deposition, disrupting the dynamic equilibrium. Thanks to the catalytic effect of EDCO, a fast desolvation process occurs. In comparison to the PE with a substantial increase of active water molecules in the IHP, the partially adsorbed EDCO nanoparticles are initially anchored on the Zn surface, regulating the local electric field and creating a uniform spatial electric field to inhibit dendrite growth. Furthermore, EDCO nanoparticles modulate the hydrogen bonding network and solvation structure in the electrolyte when accelerating Zn^2+^ desolvation, offering significant benefits for dendrite suppression and overall electrochemical performance.

**Figure 3:**
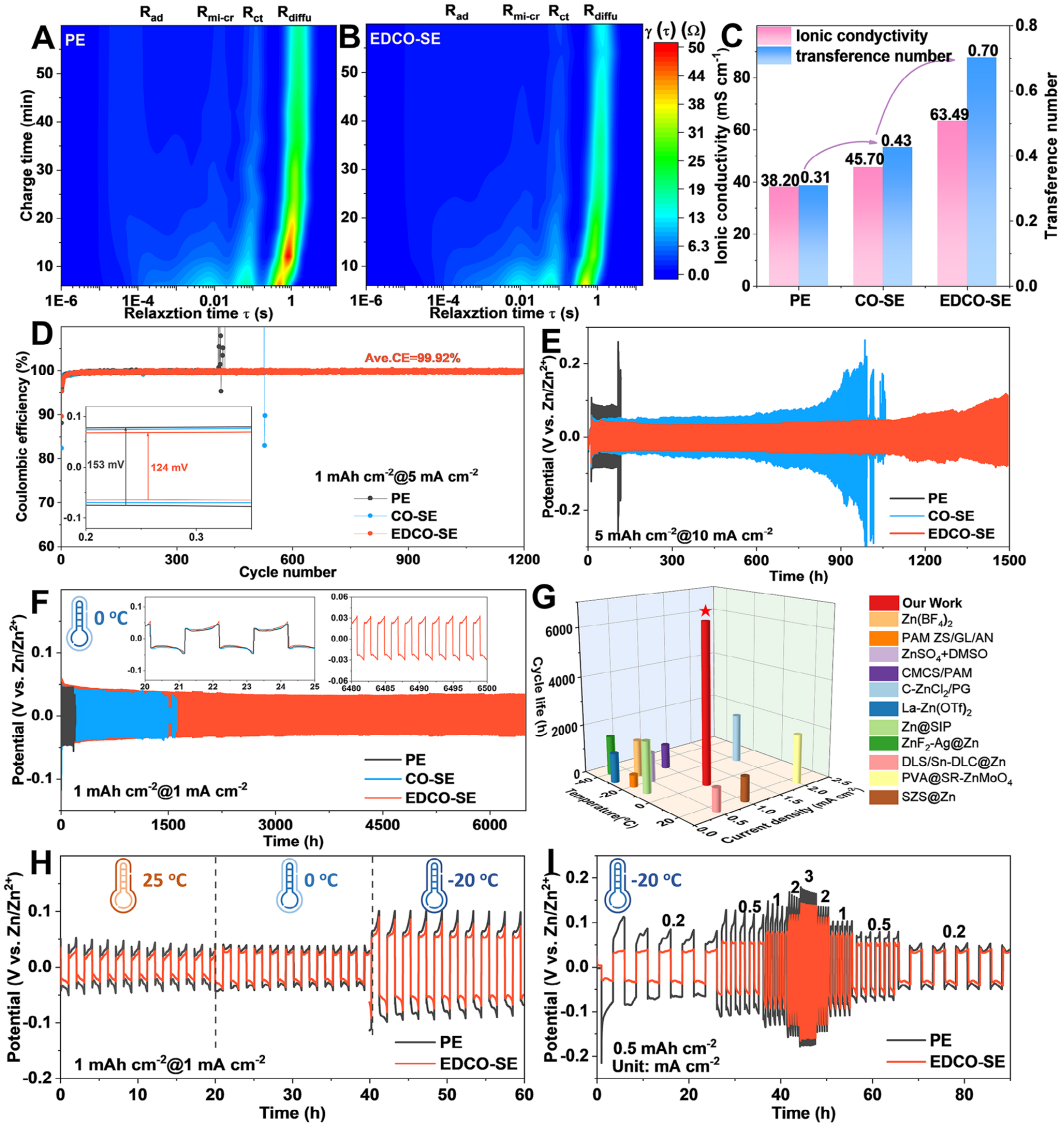
Enhanced electrochemical performance of Zn‐Zn and Zn‐Cu cells by adding EDCO. DRT analysis of Zn symmetric cells using A) PE and B) EDCO‐SE. C) Ionic conductivity and Zn^2+^ transference number of various electrolytes. D) CE values of Zn plating/stripping. E) Long‐term galvanostatic charge/discharge curves at 5 mA cm^−2^ for 10 mAh cm^−2^. F) The galvanostatic cycling of Zn symmetric cells at 0 °C. G) Comparison of the long‐term galvanostatic performance at different current densities and temperatures from our work and the reported works on AZMBs. H) The galvanostatic cycling of Zn symmetric cells at different temperatures. I) Rate performance at −20 °C.

Afterward, the electrochemical stability windows of PE and EDCO‐SE were investigated. The EDCO‐SE exhibits an extended electrochemical stability window from −0.11 to 2.50 V and a low response current within the range of −0.5–3.0 V (Figure S15, Supporting Information). The interfacial kinetics were further compared in **Figure** **3**A,B through the distribution of relaxation times (DRT) and in situ EIS analysis.^[^ ^34^ ^]^ Four distinct peaks were identified, corresponding to the adsorption of Zn^2+^ (R_ad_), migration of Zn element and the subsequent crystal Zn formation process (R_mi‐cr_), the charge transfer process of Zn^2+^ cross the interface (R_ct_), and the diffusion of Zn^2+^ within the porous Zn deposition electrode, respectively.^[^ ^35^ ^]^ During the Zn plating process, the electrochemical impedance values in PE are higher and exhibit more drastic variations than those in EDCO, indicating unstable interfacial reactions (Figure S16, Supporting Information). After 60 min, EDCO‐SE exhibits lower τ values for R_mi‐cr_, R_ct_, and R_diff_ compared to PE, indicating its significant capability to enhance the kinetics.^[^ ^36^ ^]^ Ionic conductivity and Zn^2+^ transfer number are key parameters for evaluating the electrochemical performance of batteries (Figure 3C). The EDCO‐SE exhibits a high conductivity of 63.49 mS cm^−1^, whereas the PE only has 38.20 mS cm^−1^. At the same time, the Zn^2+^ transference number is increased remarkedly from 0.31 for PE to 0.70 for EDCO‐SE (Figures S17, S18, Supporting Information). Also, the activation energy (Ea) through the Arrhenius equation is commonly employed to quantitatively characterize Zn^2+^ migration kinetics.^[^ ^37^ ^]^ The E_a_ value of EDCO‐SE was calculated to be 10.21 kJ mol^−1^, which is significantly lower than that of PE (12.79 kJ mol^−1^) (Figure S19, Supporting Information), indicating enhanced Zn^2+^ mobility affected by EDCO.

Then, the impact of EDCO‐suspension electrolyte on the reversibility of Zn plating/stripping was systematically evaluated. In Zn||Cu asymmetric cells, the EDCO‐SE demonstrated exceptional stability and a high average CE of 99.92% over 1200 cycles, compared to only 400 cycles for PE (Figure 3D). The overpotential in the EDCO‐SE was significantly reduced from 153 mV of PE to 124 mV, highlighting the enhanced Zn^2+^ transfer kinetics (Figure S20, Supporting Information). Compared with the reported results, the EDCO‐SE shows a remarkable effect on the improvement of cycle life and CE (Figure S21 and Table S1, Supporting Information). In the Zn symmetric cells, the EDCO‐SE demonstrated impressive stability of 1800 h at 5 mA cm^−2^ without significant voltage jump (Figure S22, Supporting Information). Increased to 10 mA cm^−2^, the cell with EDCO‐SE achieved a prolonged lifespan of 1500 h and a reduced voltage hysteresis of 33 mV, outperforming PE (120 h, 83 mV) under identical conditions (Figure 3E). The catalytic effect becomes more effective under different current densities, as evidenced by the excellent performance summarized in Figure S23 (Supporting Information) and the rate performance in Figure S24 (Supporting Information). The stability and effectiveness of EDCO‐SE were demonstrated by the excellent reproducibility of assembled cells after resting for 24 h, displaying a negligible difference in overpotentials. And even the cells with EDCO‐SE rested for 24 h after two cycles, the overpotential of the cells based on EDCO‐SE remained unchanged before and after resting, demonstrating its stability and capability to suppress side reactions (Figure S25, Supporting Information).

Reducing to low temperatures, the [Zn(H_2_O)_6_]^2+^ solvation shell would expand, resulting in a high desolvation barrier, which in turn leads to poor cycling stability and a short lifetime.^[^ ^38^ ^]^ It is essential to verify the functionality of the EDCO‐SE at temperatures under 0 or −20 °C. As demonstrated in Figure S26 (Supporting Information), the cell with EDCO‐SE exhibits superior rate performance at current densities ranging from 0.5 to 10 mA cm^−2^ and it exhibits much lower overpotentials than that of the PE. Thanks to the EDCO‐induced reduction of the desolvation barrier and acceleration of Zn^2+^ diffusion kinetics, the cell with suspension electrolyte exhibits an impressive long‐cycle stability of 6500 h with lower overpotentials of 34 mV (Figure 3F). The slight increase of initial overpotentials may be attributed to that the EDCO‐SE costs some time to dynamically form a stable interfacial layer on the Zn surface under low temperatures. However, after plating/stripping for 20–25 h, the overpotential of EDCO‐SE was already lower than that of PE and CO‐SE, which clearly indicated that EDCO accelerated the ion transport kinetics. Strikingly, the symmetric cell with EDCO‐SE achieves high cycling stability among the reported AZMBs from electrolytes to artificial layer engineering (Figure 3G; Table S2, Supporting Information). To further validate this strategy under more extreme conditions, the zinc trifluoromethanesulfonate (Zn(OTf)_2_) electrolyte instead of ZnSO_4_‐based electrolyte was performed at −20 °C, which is attributed to that the pristine ZnSO_4_‐based electrolyte is hard to reach such lower temperature surrounding since it will be solidified.^[^ ^32^ ^]^ Under different temperature gradients, the cell with EDCO‐SE could operate stably with a low overpotential as the temperature is decreased from 25 to −20 °C (Figure 3H). At −20 °C, the symmetric cell with EDCO‐SE achieved a small overpotential of 37 mV at 0.2 mA cm^−2^ and 140 mV at 3 mA cm^−2^ (Figure 3I), respectively. This improved performance is attributed to the enhanced conductivity that EDCO effectively promotes the transport kinetics of Zn^2+^ from 6.49 to 11.07 mS cm^−1^ under −20 °C (Figure S27, Supporting Information). Enhanced to 1 mA cm^−2^, the Zn symmetric cell with the EDCO‐SE provides remarkable stripping/plating stability for over 450 h with a stable overpotential (67 mV) (Figure S28, Supporting Information). To further broaden our strategy under the lower temperature of −30 °C, we incorporated EDCO nanoparticles into previously reported low‐temperature electrolyte formulations (2‐propanol composite electrolyte).^[^ ^39^ ^]^ The EDCO‐SE also exhibits a lower overpotential compared to the original pristine electrolyte formulation. Under a current density of 0.3 mA cm^−2^, the role of EDCO becomes more pronounced, as the Zn symmetric cell with EDCO exhibits excellent stability and a consistent overpotential of 122 mV, much lower than the controlled one (Figure S29, Supporting Information). In short, the suspension nanoparticle is unable to change the freezing point of the basic electrolyte, but it is capable of altering the solvation shell structure and desolvation pathway for fast desolvation, guaranteeing the successive ion transfer and diffusion kinetics, enabling the uniform deposition under low temperatures. These findings also confirm that the EDCO with Schottky defects in the suspension electrolyte significantly lowers the desolvation barrier and accelerates Zn^2+^ transfer kinetics.

**Figure 4:**
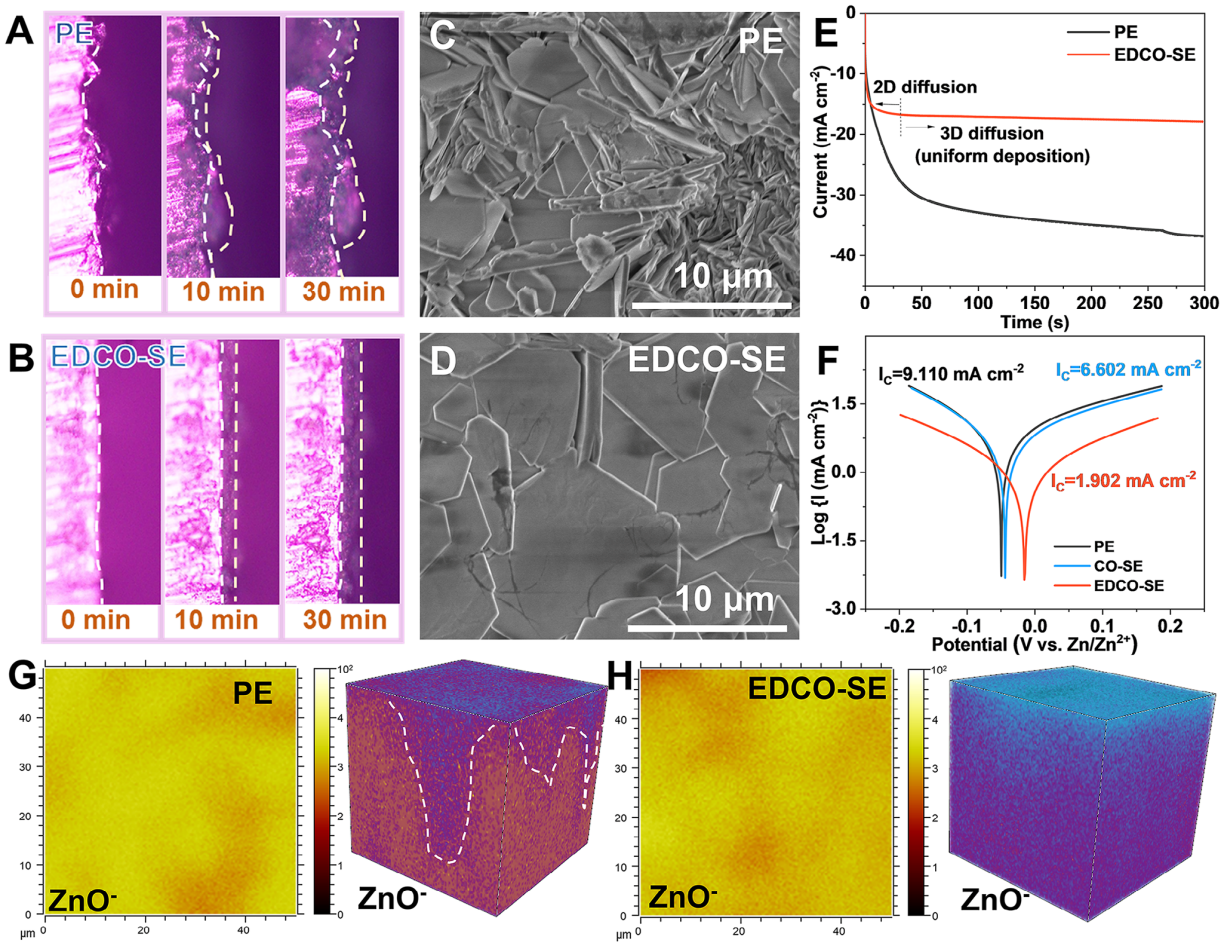
Inhibited self‐corrosion and promoted uniform Zn deposition by adding EDCO. In situ optical microscopy observation of Zn deposition in the A) PE and B) EDCO‐SE. SEM images of the plated Zn on the Cu substrate with a capacity of 10 mAh cm^−2^ in C) PE and D) EDCO‐SE. E) CA curves of the Zn anode tested in PE and EDCO‐SE at the constant potential of ‐150 mV. F) The Tafel curve for different electrolytes. 2D and 3D spatial distribution of ZnO^−^ species of Zn anode after 30 cycles in G) PE and H) EDCO‐SE via TOF‐SIMS.

The modification of EDCO on Zn deposition morphology was comprehensively evaluated using in situ optical microscopy, scanning electron microscopy (SEM), and TOF‐SIMS. As depicted in **Figure** **4**A,B, in the pristine electrolyte, the Zn surface exhibits uneven nucleation within 10 min at 1 mA cm^−2^, followed by pronounced dendritic growth over time. However, the EDCO‐SE facilitates dense and uniform deposition due to the fast desolvation kinetics and localized electric field redistribution in the micro‐space provided by EDCO. Significant differences in the SEM images of the Zn deposition morphologies on the Cu substrate were also assessed. The Zn anode in PE presents a rugged surface covered with irregular protrusions and dendrites (Figure 4C). However, the Zn anode in the suspension electrolyte exhibits a relatively smooth and dense morphology. The oxygen defects in EDCO disrupt charge balance and induce local space charge polarization, regulating ion flux and promoting uniform Zn deposition (Figure 4D). Owing to the fast desolvation, the reduction of active water molecules at the IHP effectively suppressed the HER and corrosion reactions. Chronoamperometry (CA) measurements highlighted the fast nucleation and steady 3D diffusion in the suspension electrolyte, indicating superior desolvation/diffusion kinetics (Figure 4E). The Tafel curve shows a substantial reduction in corrosion current density from 9.110 mA cm^−2^ of PE to 1.902 mA cm^−2^ of EDCO‐SE, accompanied by a positive corrosion potential shift (Figure 4F). As reconstructed in Figure 4G,H, the TOF‐SIMS imaging further confirmed the homogeneous and crack‐free morphology of the Zn anode cycled in the suspension electrolyte compared to the irregular and damaged surface in PE. The small amount of CeO_2_^−^ species on the surface of the Zn anode indicates that EDCO is not fully deposited but remains suspended in the electrolyte (Figure S30, Supporting Information). In short, the electronic delocalization and catalytic properties of EDCO introduced abundant active sites at the electrode/electrolyte interface, modulating the local electric field and enabling uniform Zn flux.

**Figure 5:**
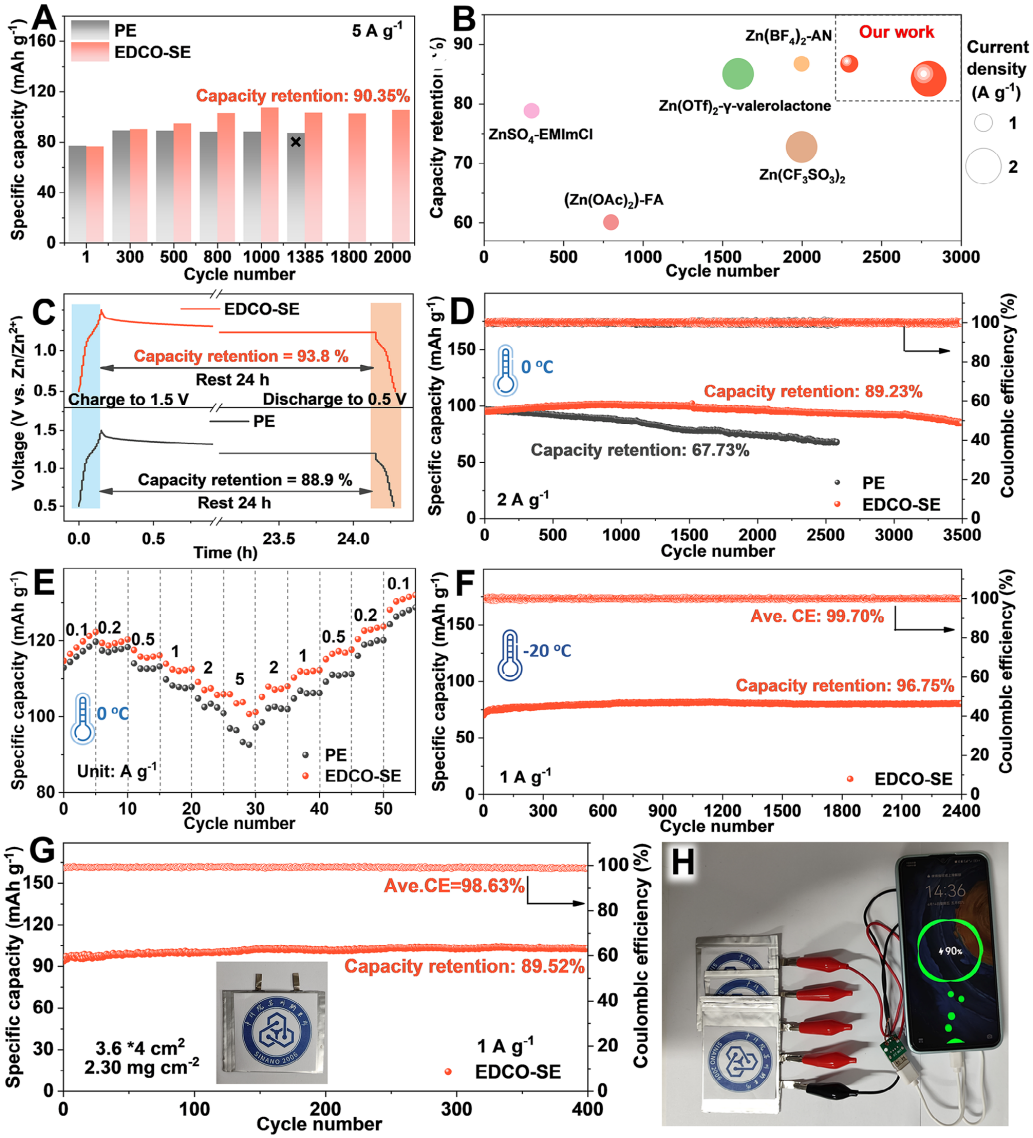
Electrochemical performance and characterization of Zn||PANI full cells. A) cycling performance of full cells at 25 °C. B) Comparison of the cycling performance at different current densities from our work and the reported works. C) Self‐discharge situation of a full cell under resting for 24 h. D) Cycling and E) rate performance of full cells at 0 °C. F) cycling performance of full cells at −20 °C. G) Cycling performance of pouch cell at 1 A g^−1^. H) Photographs of pouch cells for powering the mobile phone.

To comprehensively evaluate the performance of EDCO‐SE in full cells, **Figure** **5**A displays that the full cell with EDCO‐SE exhibits remarkable cycling stability of 2000 cycles at 5 A g^−1^, maintaining the capacity retention of 90.35%. In contrast, the PE cell experiences rapid capacity decay after 1300 cycles. The EDCO‐SE demonstrates the same stable trends under various current densities (Figures S31–S33, Supporting Information). This outstanding cycling performance is superior to that reported in the literature (Figure 5B; Table S3, Supporting Information). After cycling, the surface of the Zn anode was inspected via SEM and the Zn in the EDCO‐SE exhibits a uniform and dense Zn deposition layer without dendrites in contrast to the Zn anode in PE (Figure S34, Supporting Information). After resting for 24 h, the cell with EDCO‐SE retains 93.8% of its initial capacity compared to 88.9% of the cell with PE, suggesting the inhibition of side reactions (Figure 5C). Even under 0 °C, the cell with EDCO‐SE shows excellent cycling performance at 1 A g^−1^ and it could keep the lifespan up to 1900 cycles with a capacity retention of 99.43% (Figure S35, Supporting Information). As displayed in Figure 5D, the capacity retention of the EDCO‐SE cell remains as high as 89.23% after 3500 cycles, compared to only 67.73% for the PE cell at 2 A g^−1^. Even at a high current of 5 A g^−1^, the full cell with EDCO‐SE also demonstrates excellent stability and reversibility, whereas the PE exhibits a rapid capacity decline (Figure S36, Supporting Information). As displayed in Figure 5E, the rate performance demonstrates that the cell with EDCO‐SE retains 103.5 mAh g^−1^ at 5 A g^−1^, significantly higher than that achieved in the PE (96.4 mAh g^−1^). To verify the function of EDCO under extreme climatic conditions, full cell tests were conducted at −20 °C. As shown in Figure 5F, the specific capacity of the full cell with the suspension electrolyte is only slightly lower than that at room temperature, achieving a capacity retention of 96.75% after 2400 cycles. The practical feasibility of EDCO‐SE in the large areal pouch cell was validated. At a current density of 1 A g^−1^, the pouch cell achieves outstanding cycling stability with a capacity of up to 102.9 mAh g^−1^ after 400 cycles (Figure 5G), and successfully powers mobile phones, showing bright potential for application (Figure 5H). The enhanced cycling stability and dendrite suppression under low temperatures are attributed to the capability of EDCO in the suspension electrolyte for accelerating [Zn(H_2_O)_6_]^2+^ desolvation, improving Zn ion/atom transport kinetics, and suppressing side reactions of HER and corrosion.

## 3 Conclusion

In summary, the electrolyte engineering of suspension electrolyte employed with catalytic nanoparticles of EDCO is initially proposed to expedite the desolvation kinetics under low‐temperature. The domed electron‐adjustable EDCO modifies the [Zn(H_2_O)_6_]^2+^ solvation shell, reduces the desolvation barrier, and accelerates Zn^2+^ diffusion at the interface, as revealed by theoretical simulations, SFG, and TOF‐SIMS. Meanwhile, the optical and electronic investigations show the defect‐rich EDCO redistributes the localized electric field, enabling uniform zinc deposition without any side reactions of HER and corrosion. Consequently, the EDCO‐SE demonstrates outstanding electrochemical performance in achieving 1200 cycles with a CE of ≈99.92%, an impressive low voltage polarization of 33 mV (at 10 mA cm^−2^), and a long‐term lifespan of more than 6500 h even under the low temperature of 0 °C. The full cells with EDCO‐SE exhibit superior capacity retention of 89.23% after 3500 cycles at 2 A g^−1^ as well as a long lifespan of 400 cycles in a large‐areal pouch cell. Reduced the surrounding temperature to −20 °C, the full cell exhibits a capacity‐retention of 96.75% at 1 A g^−1^ after 2400 cycles. The suspension electrolyte engineering with catalytically self‐expediating desolvation kinetics significantly offers a new approach for realizing rechargeable low‐temperature batteries.

## Conflict of Interest

The authors declare no conflict of interest.

## Supporting information



Supporting Information

## Data Availability

The data that support the findings of this study are available from the corresponding author upon reasonable request.
