# Supplementary material for: Suspension Electrolytes with Catalytically Self‐Expediating Desolvation Kinetics for Low‐Temperature Zinc Metal Batteries
Source: Adv Mater. 2025 Mar 23;37(18):2501079. doi: 10.1002/adma.202501079 (PMC12051820; doi:10.1002/adma.202501079)
Supplement: Supplementary file 1 — Supporting Information [file ADMA-37-2501079-s001.docx]

Supporting Information

**Suspension Electrolytes with** **Catalytically Self-expediating Desolvation Kinetics for Low-Temperature Zinc Metal Batteries**

*Jing Dong^†^, Xiaomin Cheng^†^, Haifeng Yang, Huihua Li, Haitao Liu, Lujie Jia, Yongzheng Zhang*, Qinghua Guan, Jiqiang Jia, Fanglin Wu, Jing Zhang, Meinan Liu, Hongzhen Lin* & Jian Wang**

J. Dong, Q. Guan, Prof. M. Liu, Prof. H. Lin

School of Nano-Tech and Nano-Bionics, University of Science and Technology of China, Hefei 230026, China

E-mail: hzlin2010@sinano.ac.cn

J. Dong, X. Cheng, H. Yang, L. Jia, Q. Guan, Prof. M. Liu, Prof. H. Lin

*i*-Lab & CAS Key Laboratory of Nanophotonic Materials and Devices, Suzhou Institute of Nano-Tech and Nano-Bionics, Chinese Academy of Sciences, Suzhou 215123, China

E-mail: hzlin2010@sinano.ac.cn

H. Li, F. Wu, Dr. J. Wang

Helmholtz Institute Ulm (HIU), Ulm D89081, Germany

Karlsruhe Institute of Technology (KIT), Karlsruhe D76021, Germany

E-mail: jian.wang@kit.edu；wangjian2014@sinano.ac.cn

H. Liu

Laboratory of Computational Physics, Institute of Applied Physics and Computational Mathematics, Beijing 100088, China

Y. Zhang

State Key Laboratory of Chemical Engineering, East China University of Science and Technology, Shanghai 200237, China

E-mail: zhangyongzheng@ecust.edu.cn

Dr. J. Jia, Dr. J. Zhang

Advanced Materials Analysis and Test Center, School of Materials Science and Engineering, Xi’an University of Technology, Xi’an 710048, China

† These authors contributed to this work equally.

**Other Supplementary Materials for this manuscript:**

**Synthesis and preparation of CeO_2-_*_x_* Particles**

CeO_2_ (CO) particles were synthesized via a hydrothermal method. A buffer solution was prepared by dissolving 5 g of sodium acetate and 5 mL of acetic acid in 25 mL of deionized water, followed by stirring for 30 min. Subsequently, 5 mmol of Ce(NO_3_)_3_ was dissolved in the buffer solution, and the resulting mixture was stirred for additional 30 min. The prepared solution was then transferred into a 100 mL polytetrafluoroethylene (PTFE)-lined stainless steel autoclave and subjected to hydrothermal treatment at 200 ^o^C for 24 h. After the autoclave was cool to room temperature, the resultant precipitate was washed five times with ultrapure water, collected by centrifugation, and finally freeze-dried to obtain CeO_2_ particles. Followingly, **t**he CO particles were placed in a tube furnace and heated to 400 ^o^C for 3 h under an Ar/H_2_ flow (95 vol% Ar mixed 5 vol% H_2_). The product was removed after cooling to room temperature and recorded as electron-delocalized CeO_2-_*_x_* (EDCO).

**Preparation of Suspension Electrolyte**

The pristine aqueous ZnSO_4_-based electrolyte is set as 2 mol L^-1^. The EDCO nanoparticles are added to the above electrolyte at the concentration ranging from 1 to 10 mg mL^-1^, and the mixture is stirring until the nanoparticles are uniformly dispersed, forming a suspension electrolyte. Without any specific statement, the particle concentration in the suspension electrolyte is set as 3 mg mL^-1^. For the low temperature test, the 3 M Zn(OTf)_2_ pristine electrolyte was selected and the addition of EDCO nanoparticles is set as 3 mg mL^-1^. To decrease the freezing point, the electrolyte tested at -30 ^o^C employed with 2-propanol (30 vol%) was prepared into the 1.5 M Zn(OTf)_2_ electrolyte with the 3 mg mL^-1^ EDCO.

**Preparation of Polyaniline (PANI)** **cathodes**

2.187 mL of aniline was added to 250 mL of 1 mol L^-1^ HCl and the mixed solution was stirred for 30 min. Dissolve 5.472 g of (NH_4_)_2_S_2_O_8_ and 2 g CNTs in 250 mL of 1 mol L^-1^ HCl and gradually added dropwise to the above solution. The reaction was conducted in an ice bath for 24 h. Subsequently, it was washed three times with water and dried at 60 ^o^C to obtain PANI. The working electrode was prepared by mixing the PNAI with carbon black and polyvinylidene fluoride (PVDF) in a mass ratio of 8:1:1. The mixture slurry was coated onto the carbon film and dried at 40 ^o^C for 5 h. The mass loading of PANI in the electrode was around 1.0 mg cm^-2^.

**Electrochemical measurement**

The CR2025 coin cell employed with Zn foils with a thickness of 100 µm as the electrode and a piece of glass microfiber GF/B (Whatman) separator were assembled, where 150 µL of the corresponding electrolyte was added. The Electrochemical cycling tests of Zn symmetric cells, Zn||Cu cells, Zn||PANI cells were recorded on battery testing instruments (Land CT2001A). Electrochemical impedance spectroscopy (EIS) and linear sweep voltammetry (LSV) of coin cells were obtained on an electrochemical workstation (VMP-3, France), and EIS was performed in a frequency range of 200 kHz to 0.1 Hz at open circuit potential.

**Characterization methods**

The morphology was examined using a field-emission scanning electron microscope (SEM, Hitachi Regulus 8230). Transmission electron microscopy (TEM) and high-resolution TEM (HRTEM) were conducted using a FEI Titan G2 60-300 (FEI, USA) microscope at 300 kV to obtain structural information on the prepared nanoparticles. CO and EDCO nanoparticles were analyzed by X-ray diffraction (XRD) on a Bruker D8 Advanced diffractometer with Cu Kα radiation. X-ray photoelectron spectroscopy (XPS) were collected on an ESCALAB 250XI system to investigate the chemical surroundings of nanoparticles. The *in-situ* sum frequency generation (SFG) measurements with/without the bias voltage of 0-150 mV were performed. With the picosecond laser system, visible light wavelength was set at 532 nm and the IR pulse ranges from 3000 to 3800 cm^-1^, which were directly shone on the interface between the Zn anode and the PE/ EDCO-SE. Raman spectral analyses were performed by using a Raman spectroscope (10-1000 μm Resolution: <=0.65/cm/LabRAM HR, excited by 514 nm laser). Time-of-flight secondary-ion mass spectrometry (TOF-SIMS) was utilized to measure the three-dimensional morphology and two-dimensional distribution of cycled Zn in different electrolyte.

**Simulation methods**

All calculations in this work were carried out within the framework of the spin-polarized density functional theory, which was implemented in the *Vienna* Ab initio Simulation Package (VASP). The Perdew-Burke-Ernzerhof (PBE) functional was employed to approximate the exchange-correlation potential. The projector augmented wave (PAW) method was used to describe the ion-electron interactions. To account for the strong correlation effects induced by the 4*f* electrons of Ce, the Hubbard U correction was added to improve the results. The effective parameter U_eff_=U-J was set as 5 eV for the Ce 4*f* orbitals. Additionally, Grimme’s D3 method was adopted to rectify the deficiency of the van de Waals interaction by the PBE functional. A plane wave basis set with a cutoff energy of 600 eV was used, and the optimized lattice constant of the cubic fluorite structure was 5.475 Å, in good agreement with the experimental value. The CeO_2_(111) surface was modeled using a slab model with the (4×4) surface unit cell. The slab model consisted of four CeO_2_ tri-layers (namely twelve atomic layers). During the geometry optimization, the bottom two tri-layers were fixed, while the top two tri-layers were fully relaxed. To prevent the artificial interaction between the slab and its neighboring images, a vacuum layer of more than 15 Å was added along the z-direction. The 3×3×1 Monkhorst-Pack k-point grid was used for sampling the Brillouin zone. The convergence criteria for the energy in the self-consistent field calculation and the residual force in the geometry optimization were set at 1×10^-6^ eV and 0.03 eV Å^-1^, respectively. The adsorption energy was defined as E_ads_ = E(Zn+slab)-E(slab)-E(Zn), where the E(Zn+slab), E(slab) and E(Zn) represent the total energies of the slab with an adsorbed Zn atom, the slab itself and the isolated Zn atom, respectively.


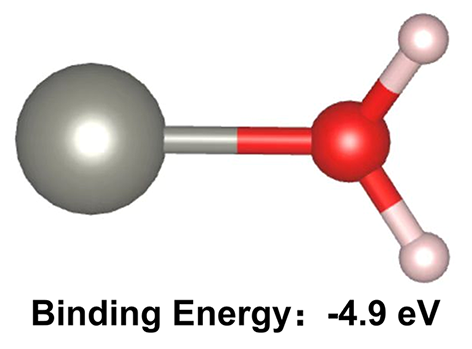


**Figure S1.** Calculated binding energy for H_2_O-Zn^2+^.


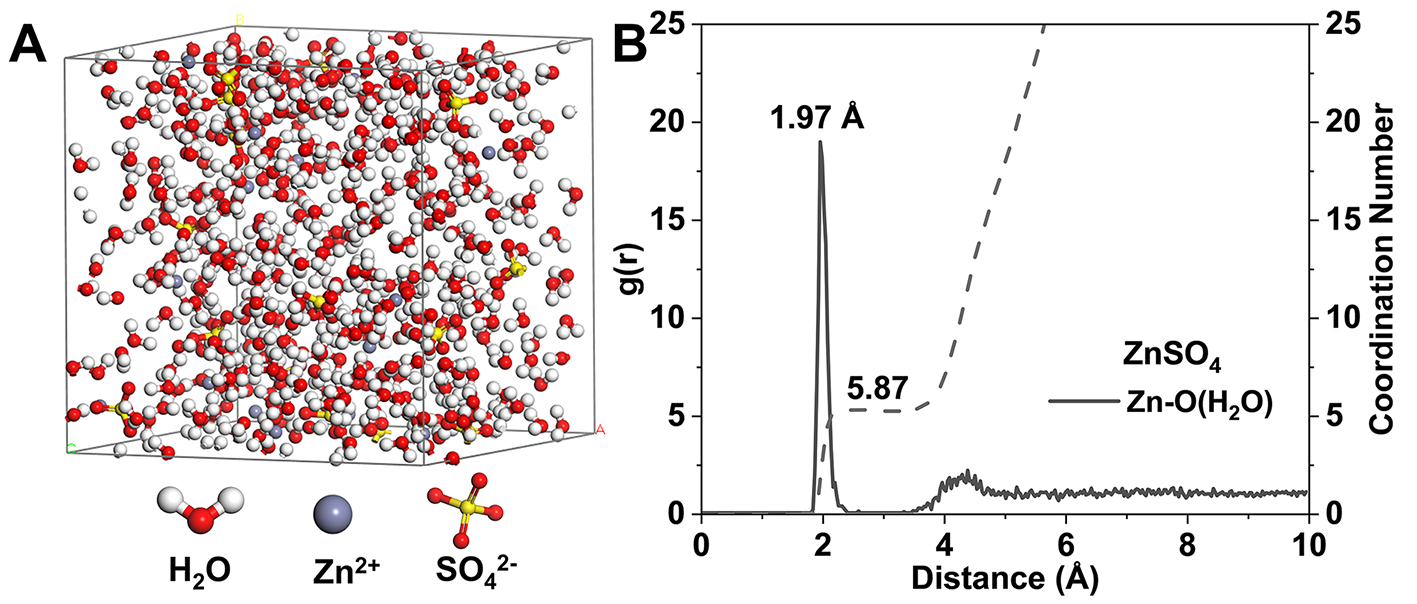


**Figure S2.** A) The 3D snapshot of the ZnSO_4_ electrolyte obtained from MD simulations; B) RDFs for Zn-O (H_2_O) in the PE.

**
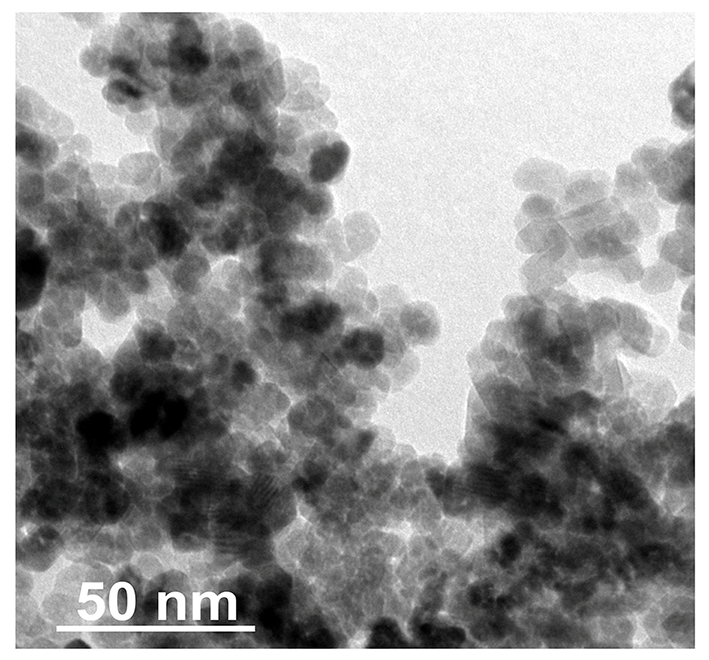
**

**Figure S3.** TEM image of EDCO.

**
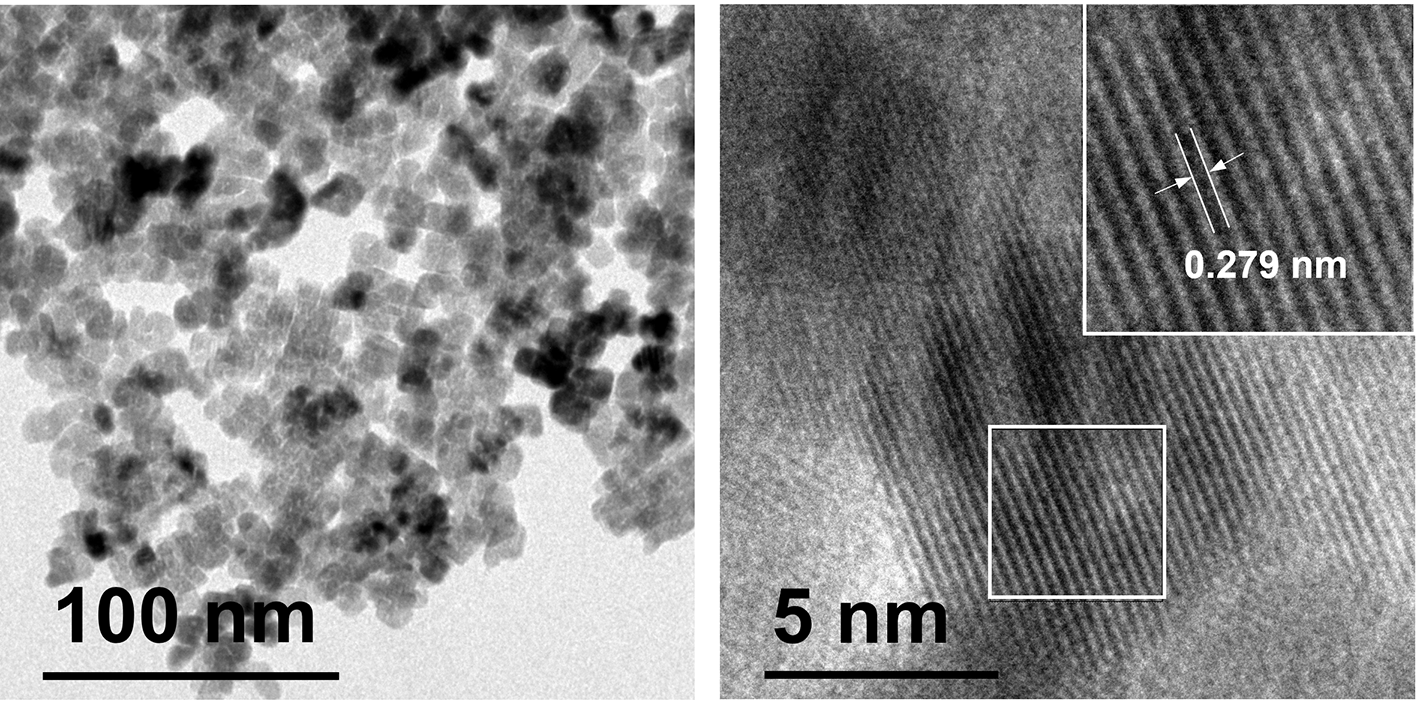
**

**Figure S4.** TEM image and HRTEM image of CO.

**
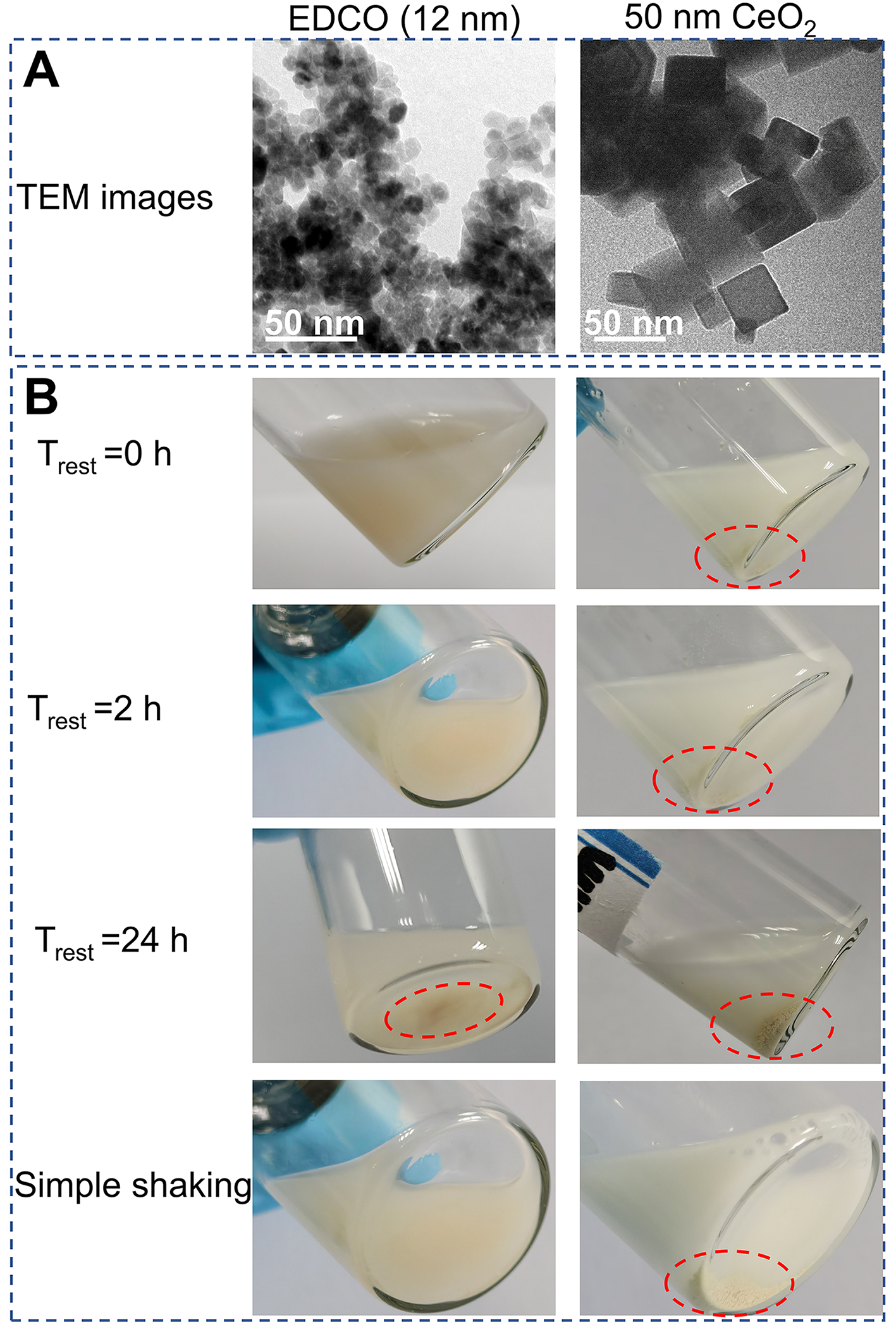
**

**Figure S5.** A) TEM images of 12 nm EDCO and 50 nm CeO_2_. B) Timeline images of the suspension electrolytes containing 3 mg mL^-1^ of EDCO nanoparticles or 50 nm CeO_2_ in 2M ZnSO_4_ electrolyte.

**
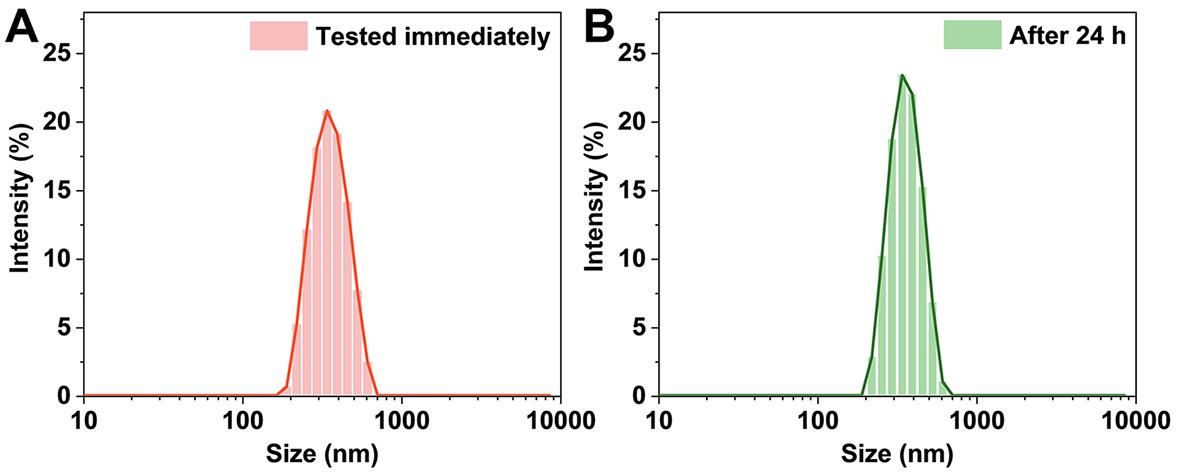
**

**Figure S6.** The EDCO particle size distribution in the SE for A) immediate testing and B) rest for 24 h after shaking.


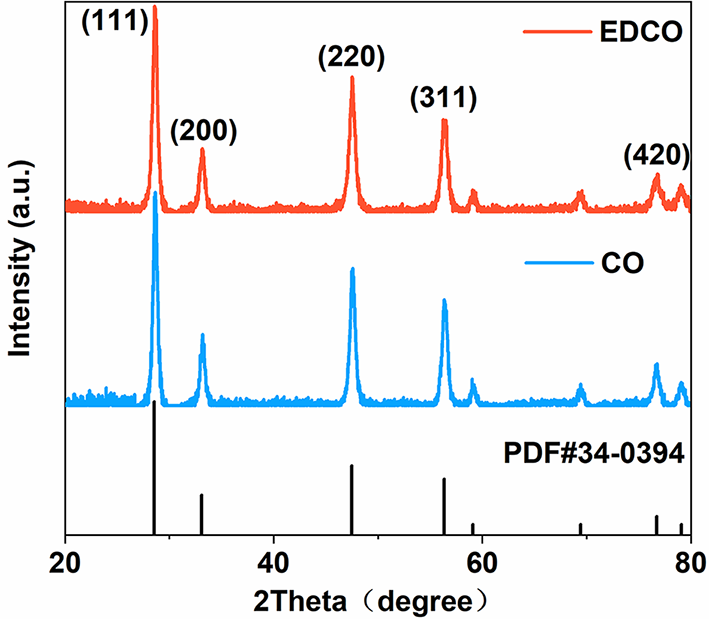


**Figure S7.** XRD patterns of the EDCO and CO nanoparticles.


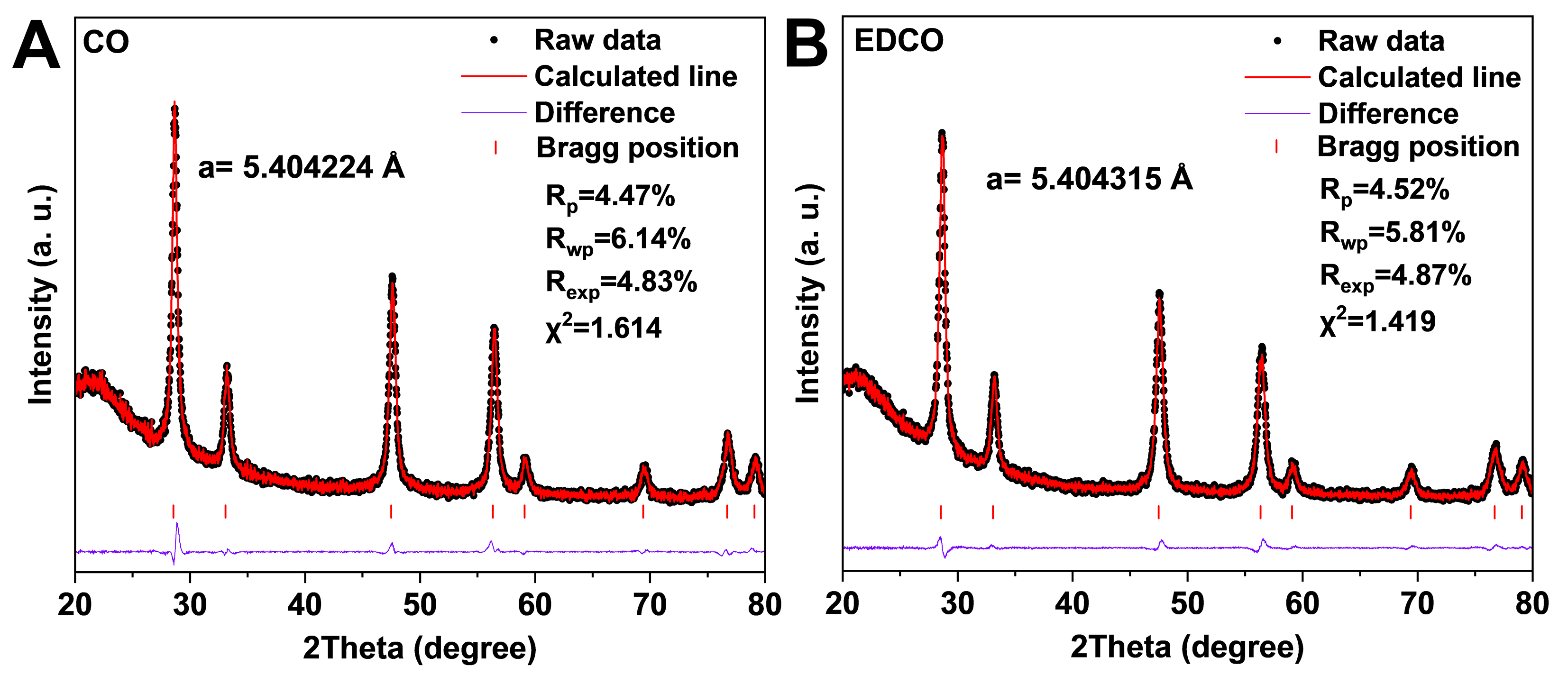


**Figure S8.** The Rietveld refinements of the XRD of A) CO and B) EDCO.





**Figure S9.** The high-resolution XPS spectra comparison of Ce 3d for EDCO and CO.

**

**

**Figure S10.** Mott-Schottky plots obtained on symmetric cells using 1 mol L^-1^ Na_2_SO_4_-CO electrolyte and Na_2_SO_4_-EDCO electrolyte at the frequency of 1 kHz.


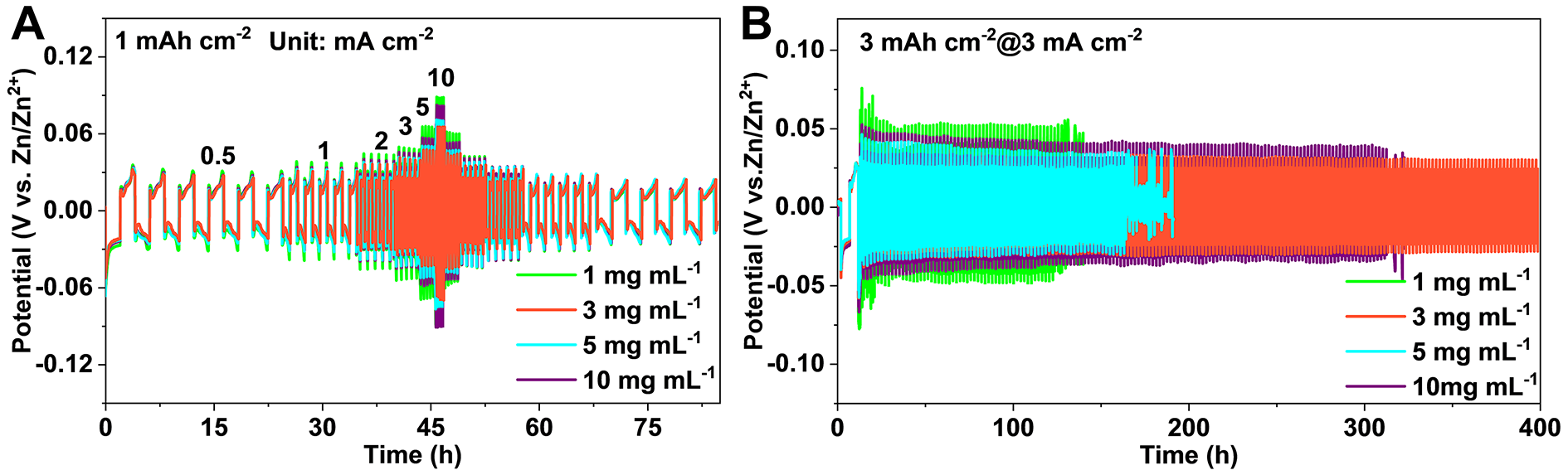


**Figure S11.** A) Rate and B) cycling performance of suspension electrolytes with various concentrations of EDCO.


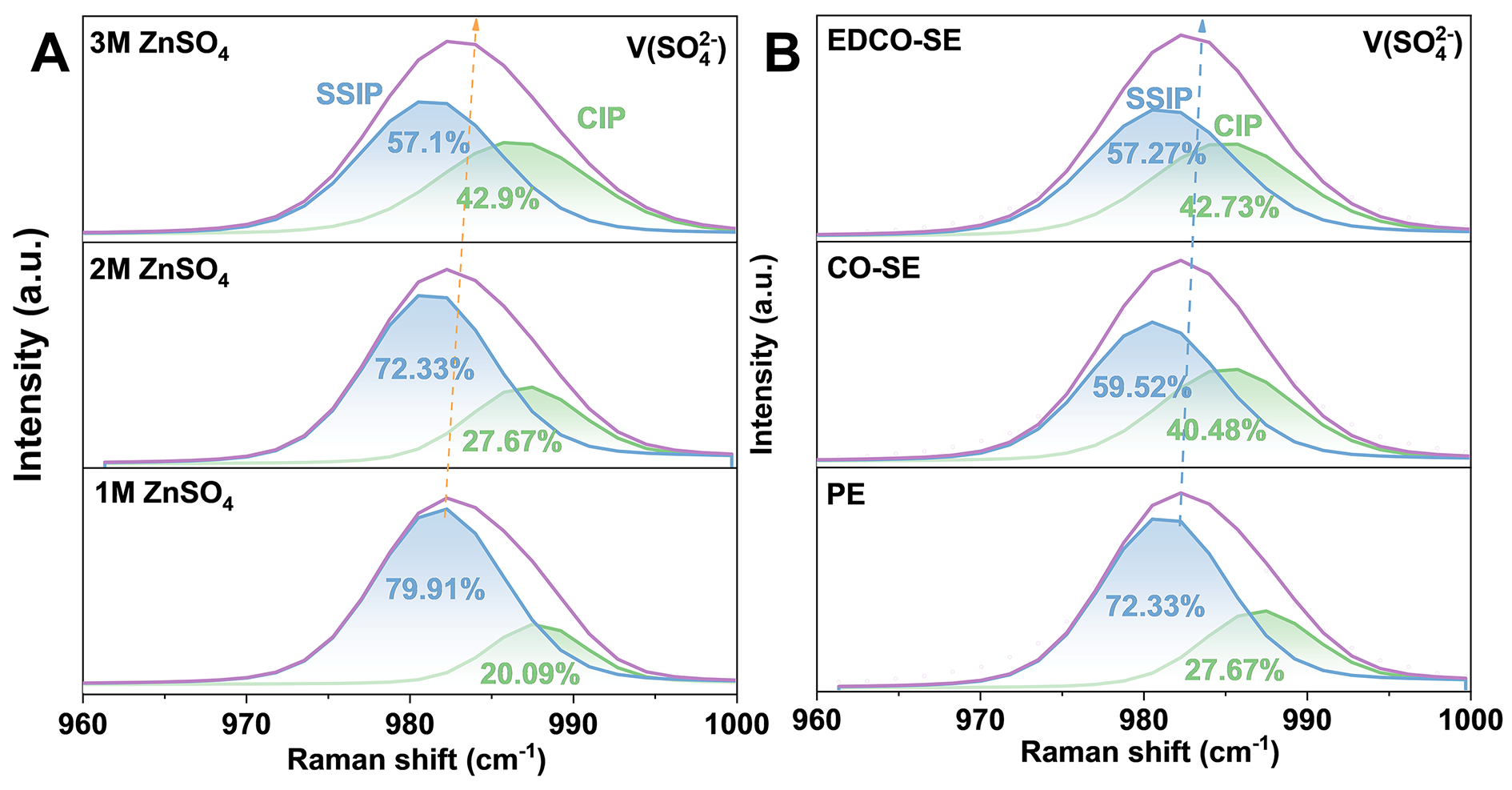


**Figure S12.** Raman signals corresponding to v(SO_4_)^2-^ of various electrolytes.


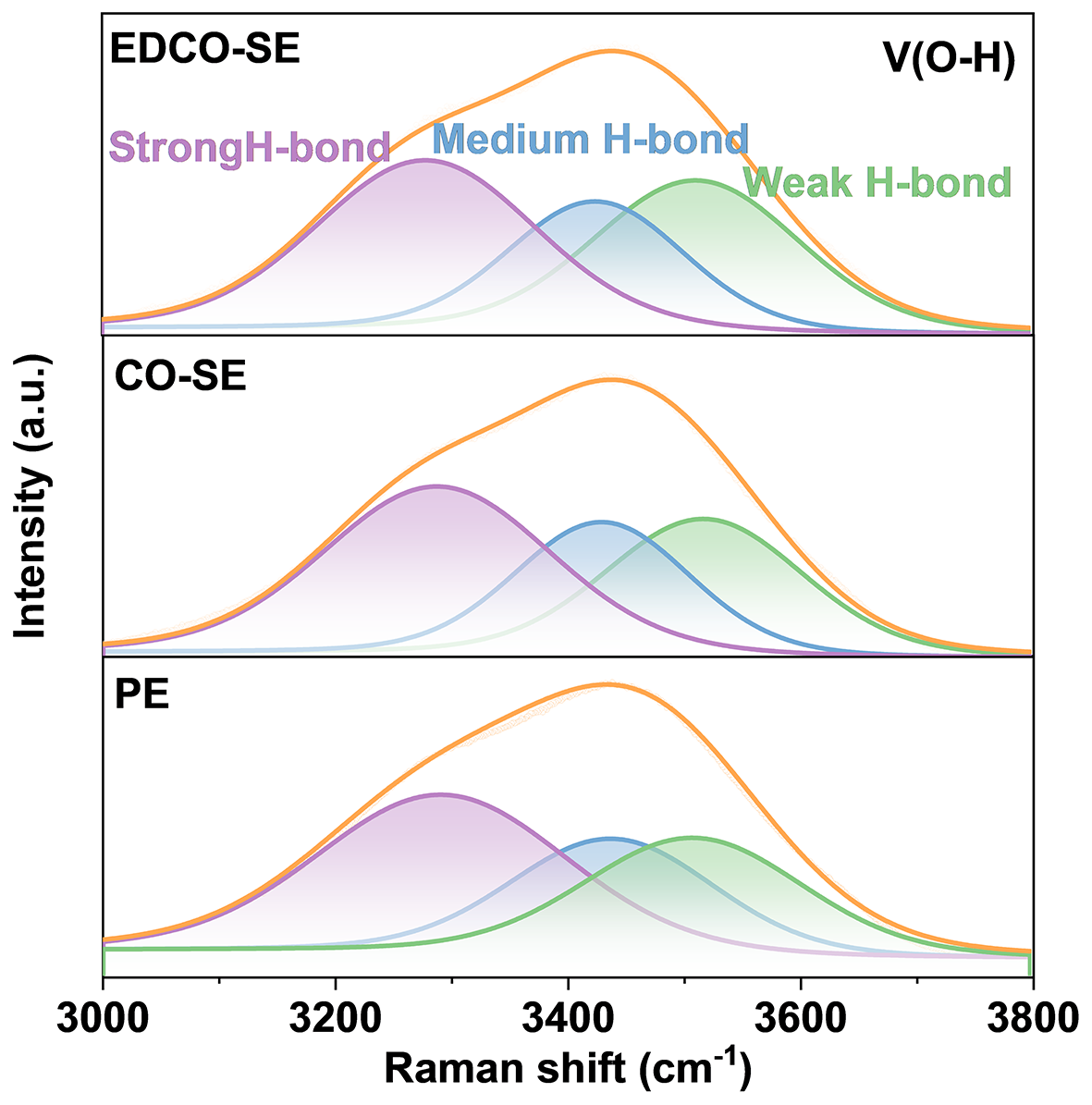


**Figure S13.** Raman signals corresponding to *v*(O-H) of various electrolytes.


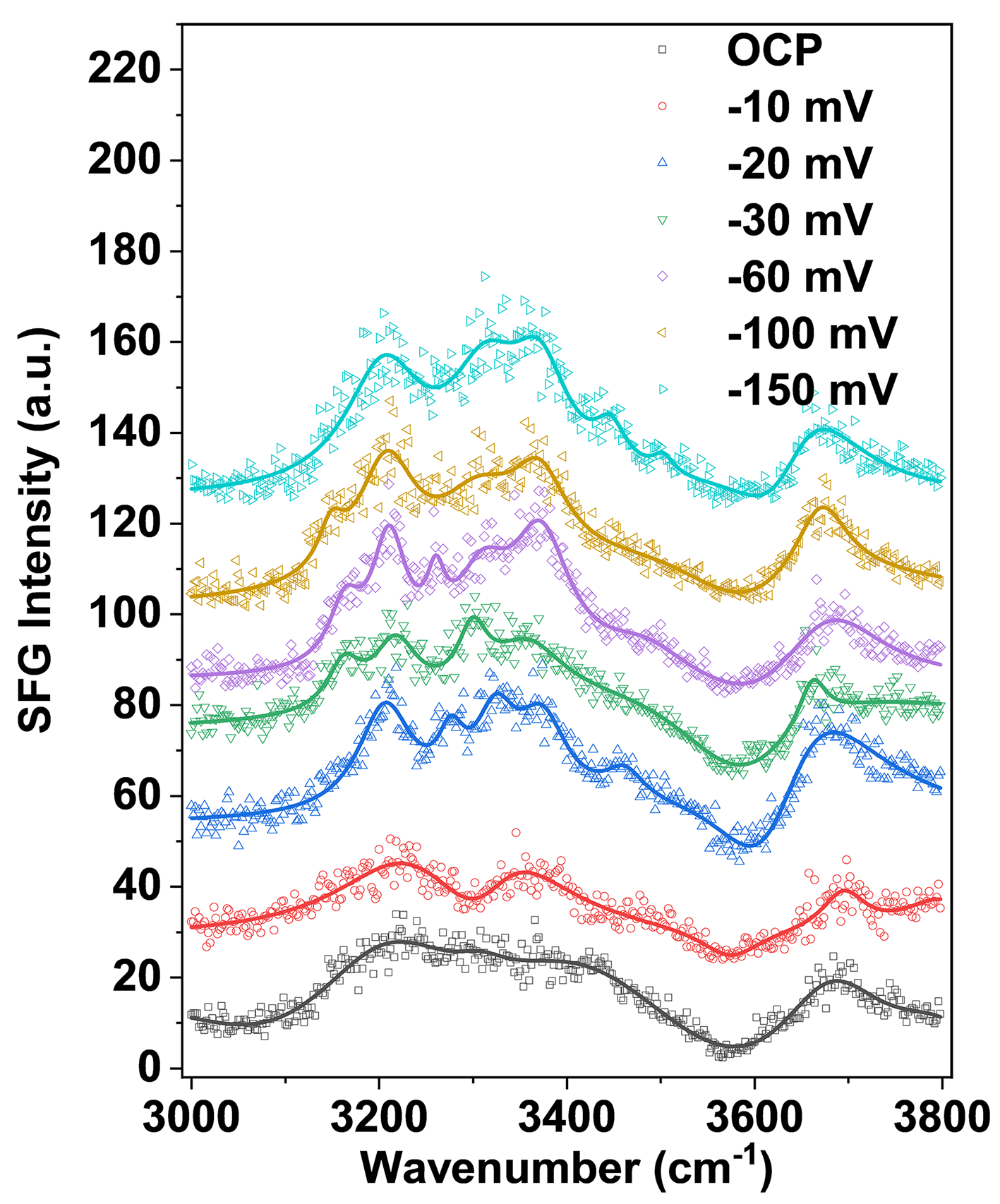


**Figure S14.** SFG-intensity changes of O-H region in PE.


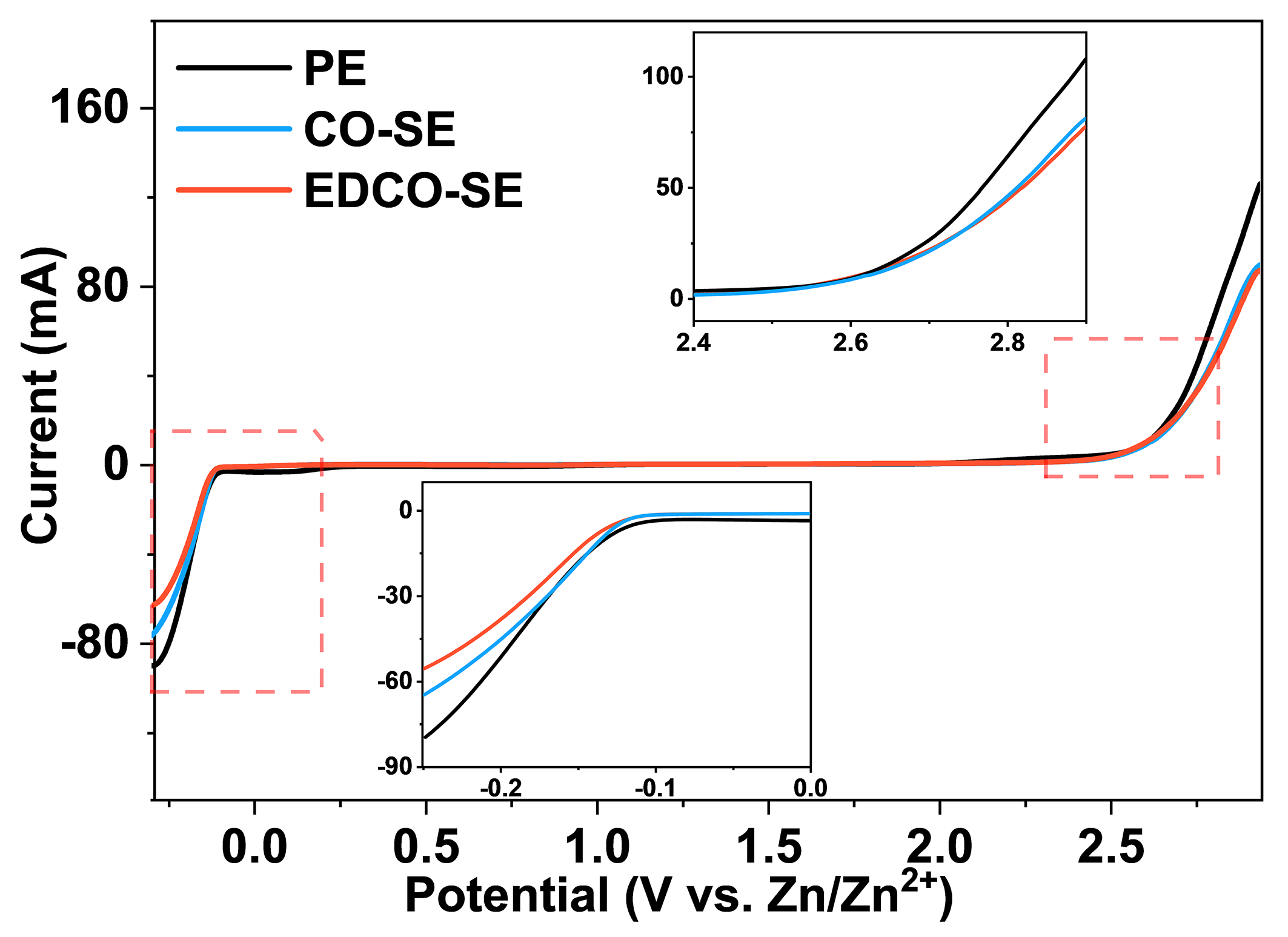


**Figure S15.** Electrochemical stability window for various electrolytes measured by linear sweep voltammetry tests.


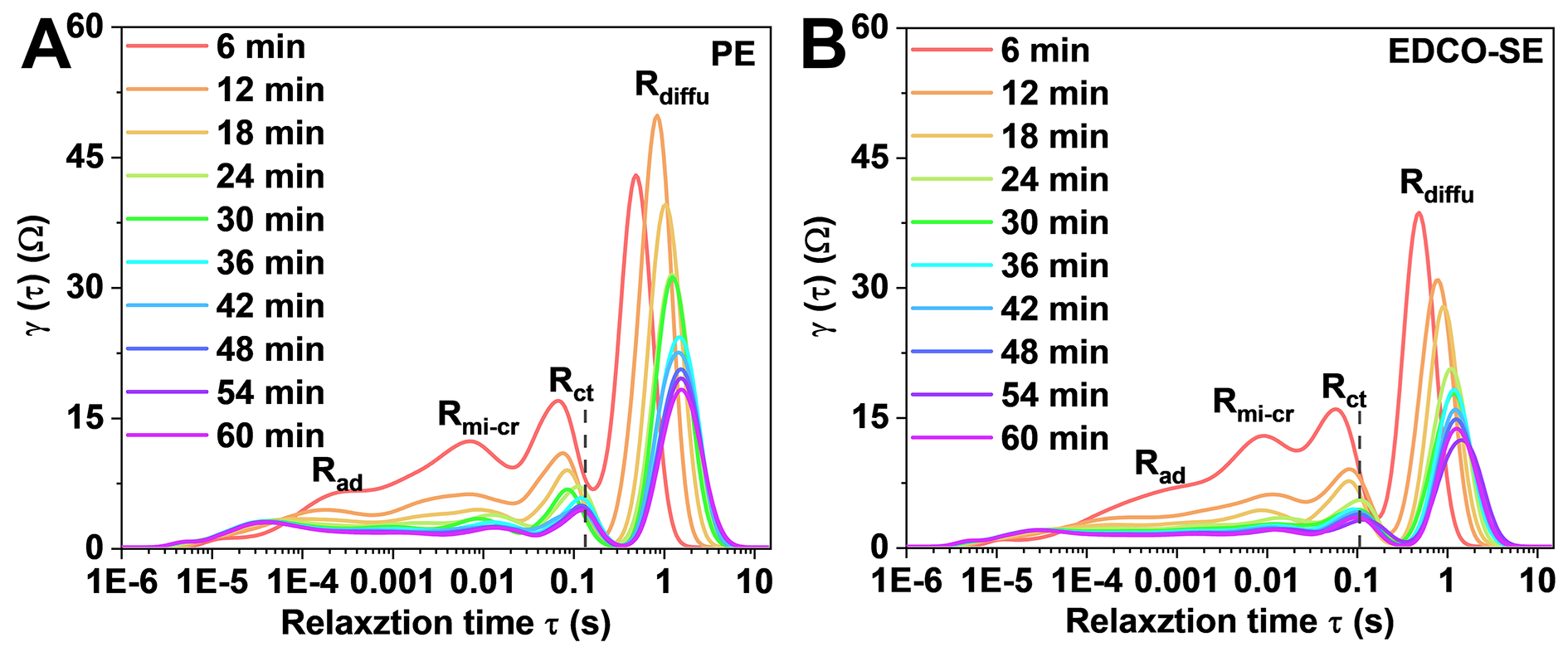


**Figure S16.** The analysis of DRT of operando EIS during Zn deposition in A) PE; B) EDCO-SE.


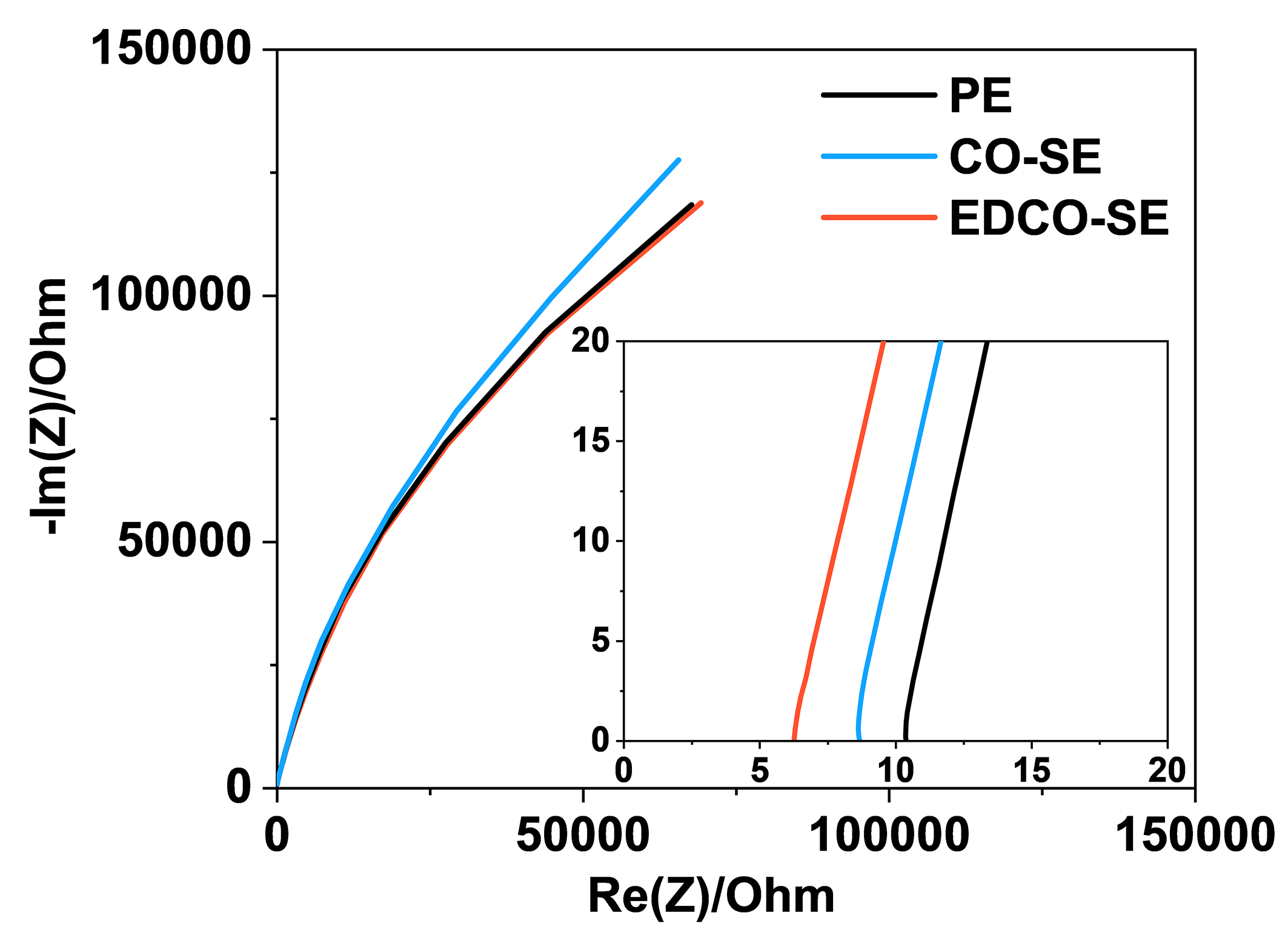


**Figure S17.** Ionic conductivity test of various electrolytes.


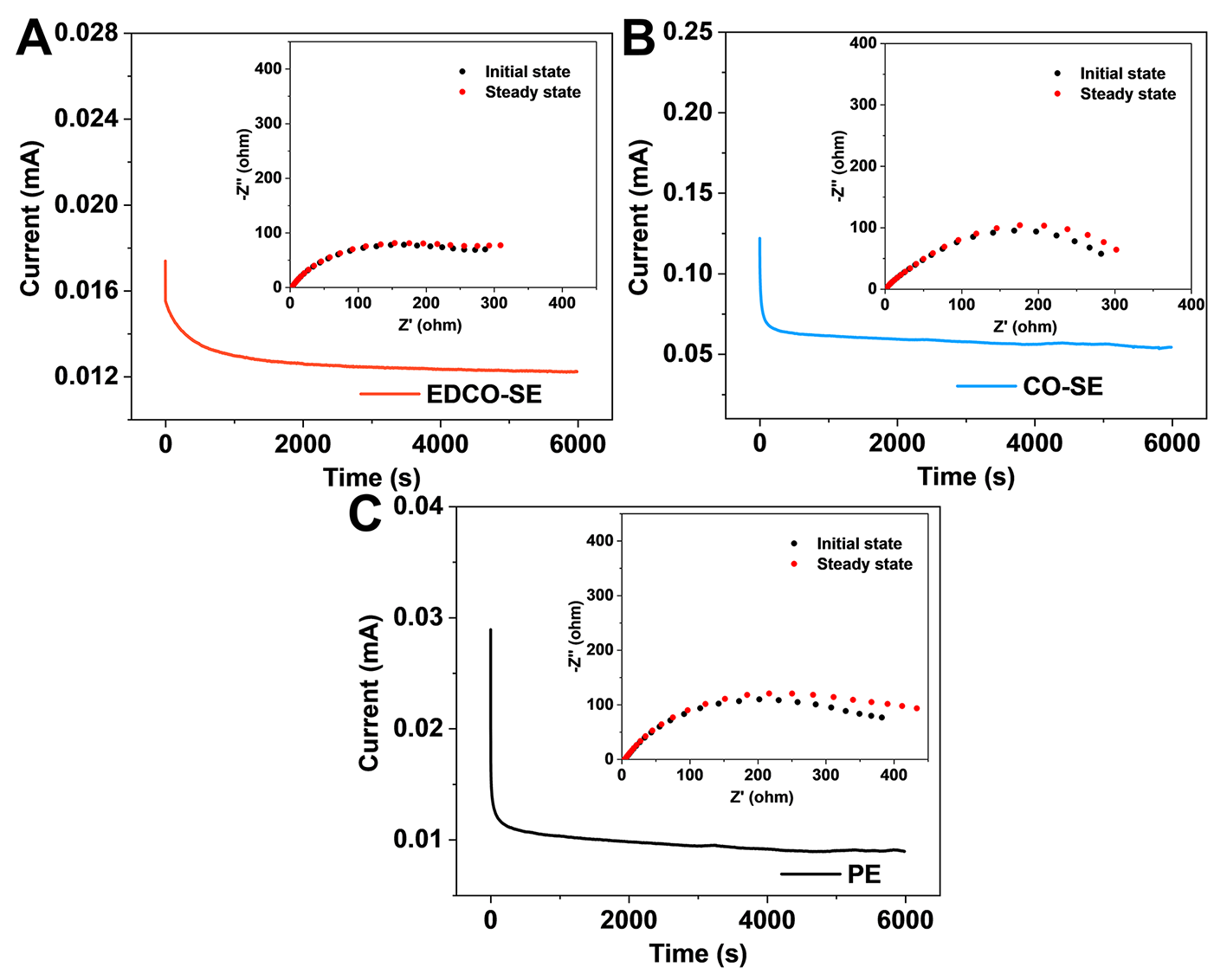


**Figure S18.** The Zn^2+^ transference number of A) EDCO-SE; B) CO-SE; C) PE at room temperature; the inset shows the EIS before and after polarization.


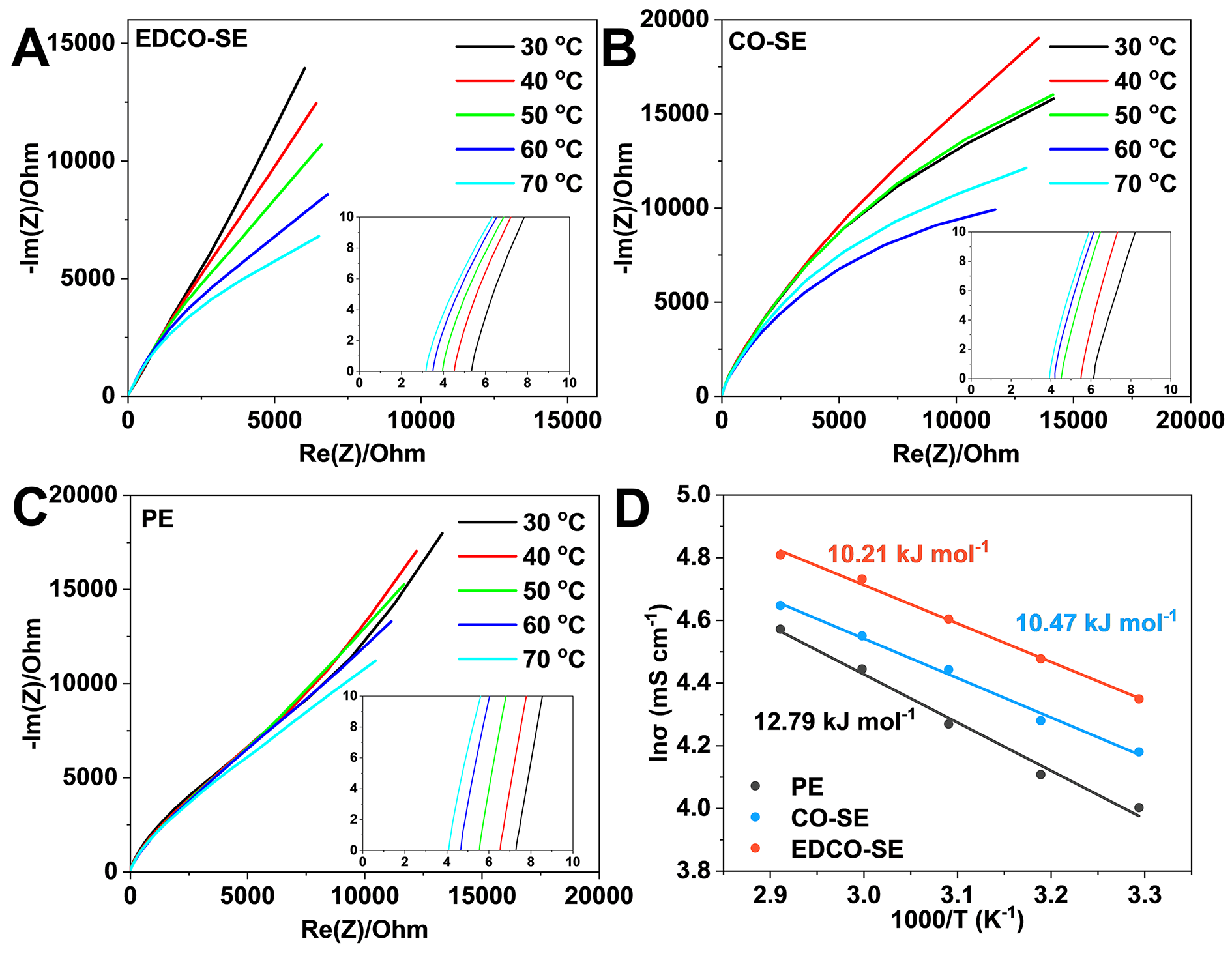


**Figure S19.** EIS curves of Zn symmetric cell at various temperatures in A) PE; B) CO-SE; C) EDCO-SE. D) The calculated activation energies of Zn^2+^ diffusion in Zn/electrolyte interface by the Arrhenius equation.


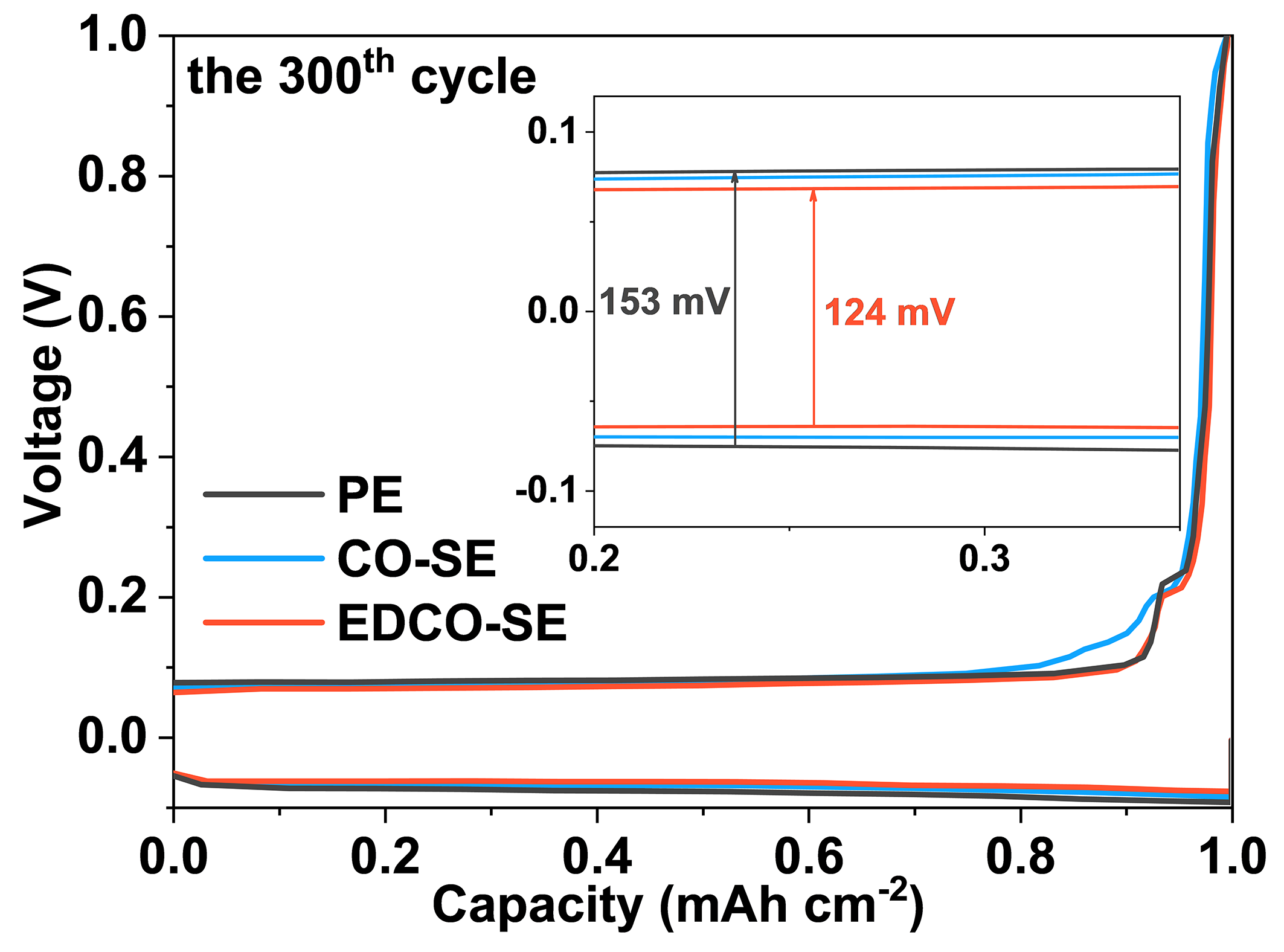


**Figure S20.** Discharge/charge voltage profiles of the asymmetrical cells in the 300^th^ cycle.


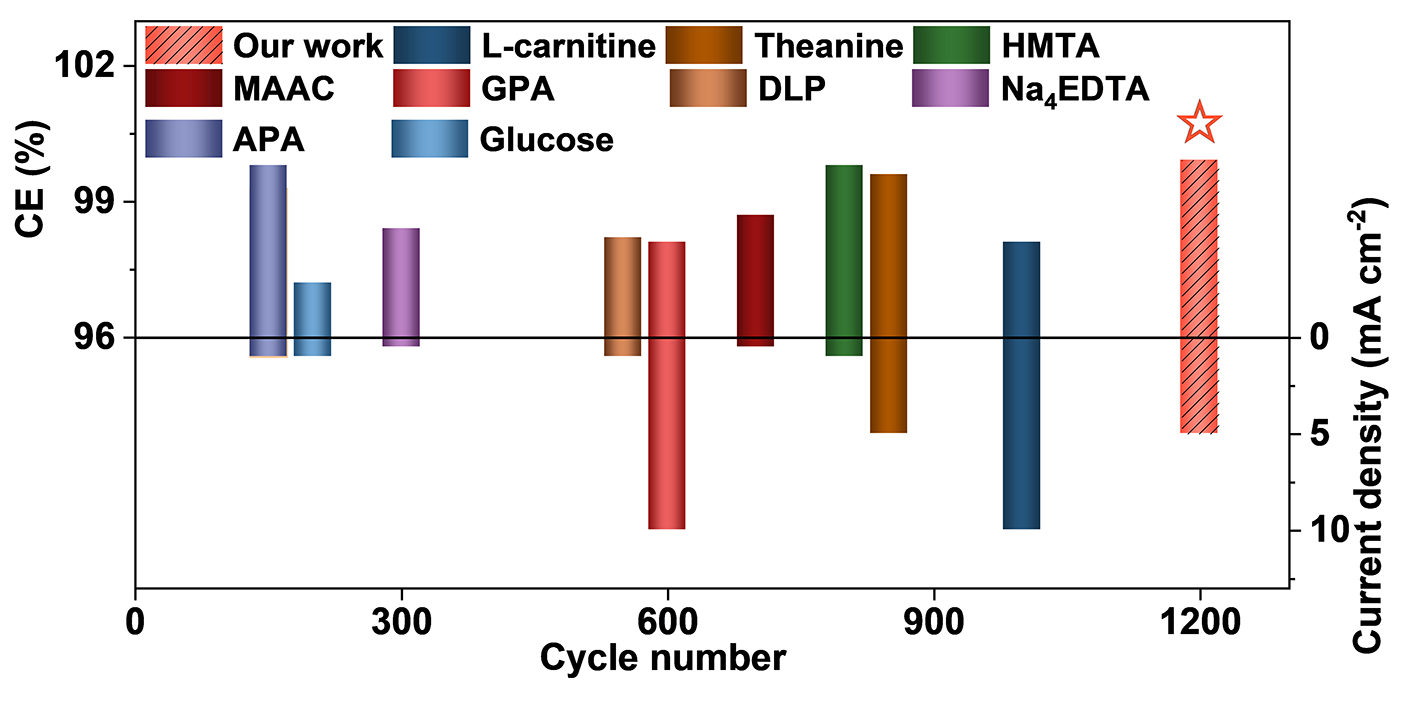


**Figure S21.** Cycle life and CE comparison with recently reported literatures utilizing different additives.


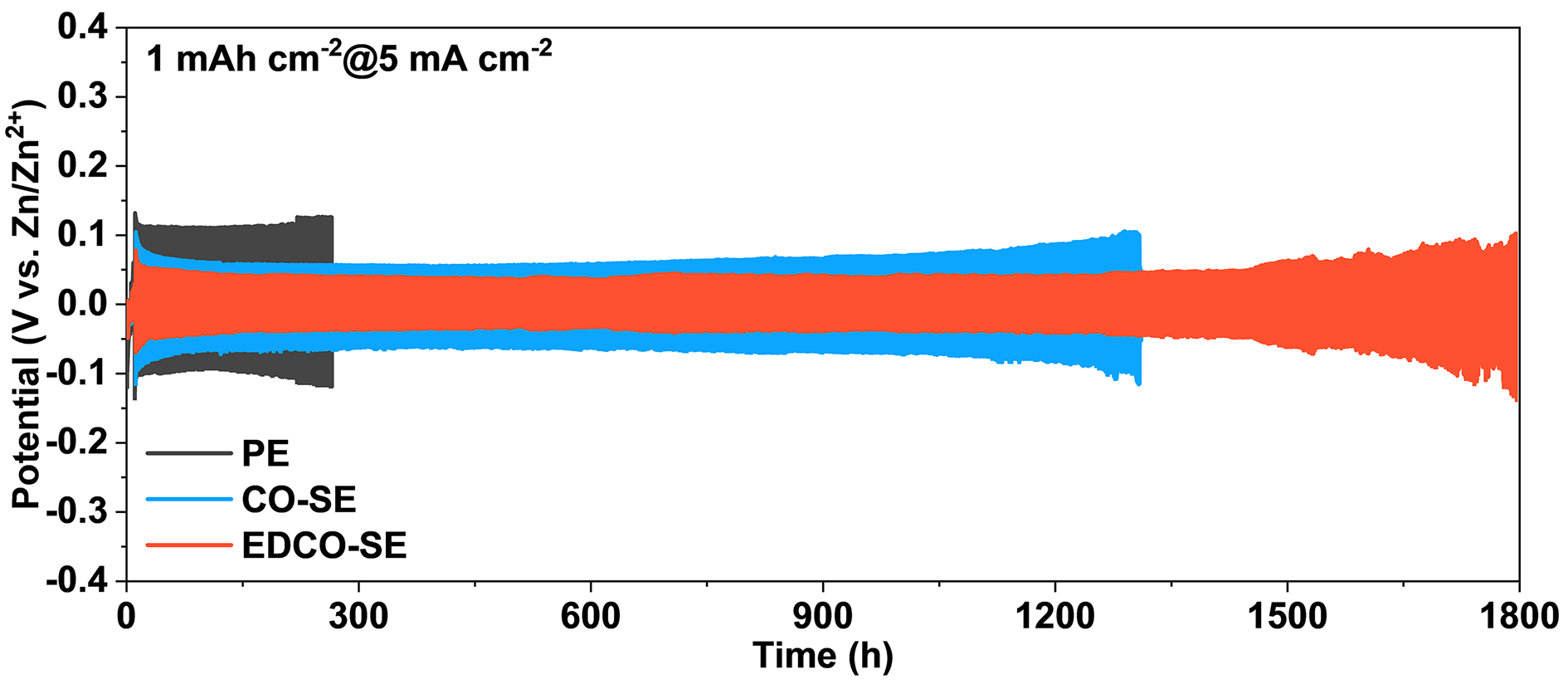


**Figure S22.** Long-term galvanostatic charge/discharge curves at 5 mA cm^-2^ for 1 mAh cm^-2^.


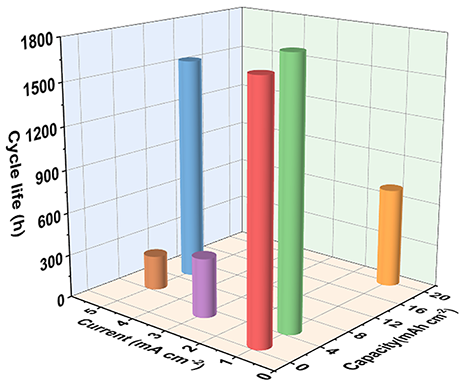


**Figure S23.** The summary of long-term galvanostatic charge/discharge performance at different current densities.


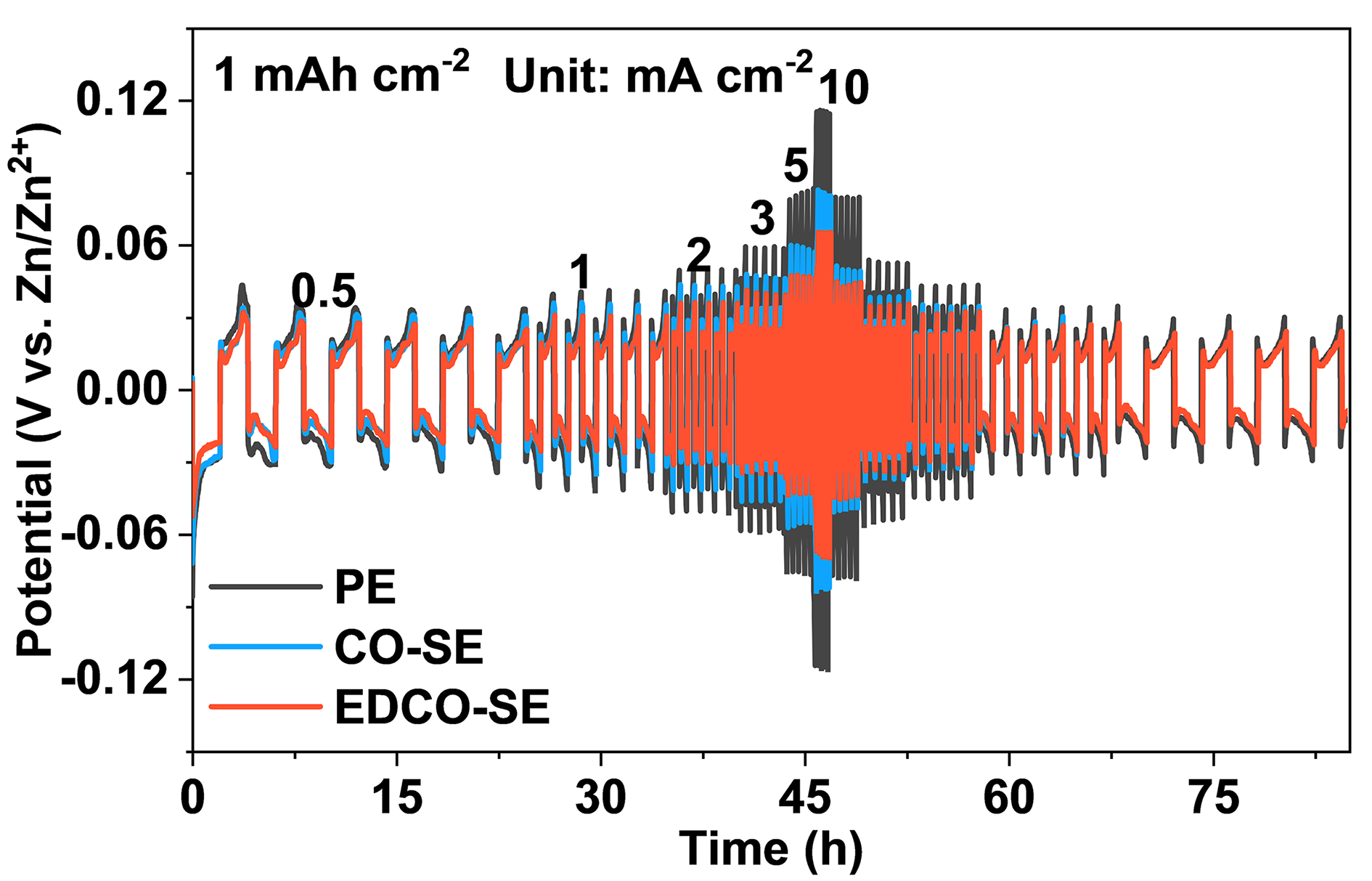


**Figure S24.** Rate performance from 0.5 to 10 mA cm^-2^.


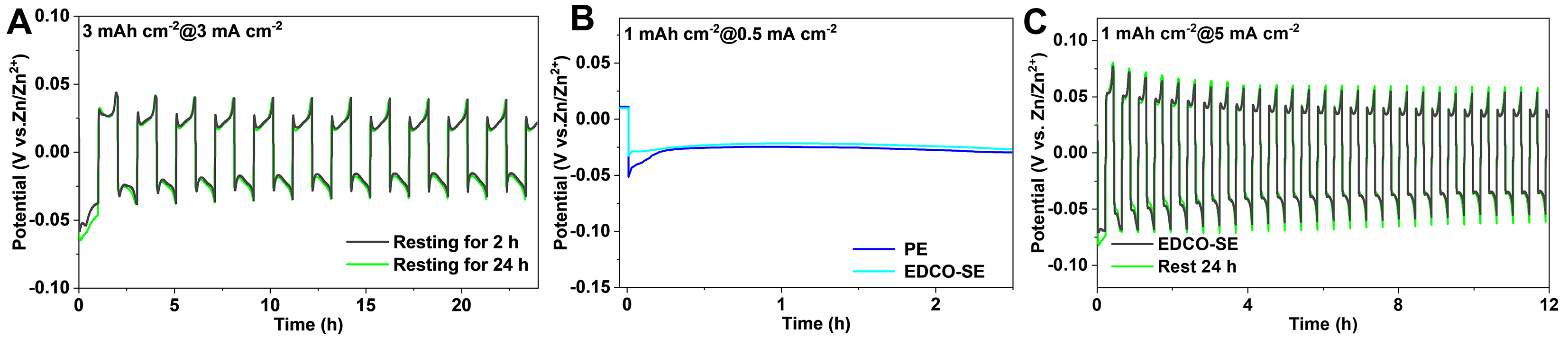


**Figure S25.** A) Charge/discharge curves of cells with EDCO-SE resting different hours after assembly at 3 mA cm^-2^ for 3 mAh cm^-2^. B) Charge/discharge curves of cells resting for 24 h after two cycles. C) Charge/discharge curves before and after 24 h of standing time of EDCO-SE.


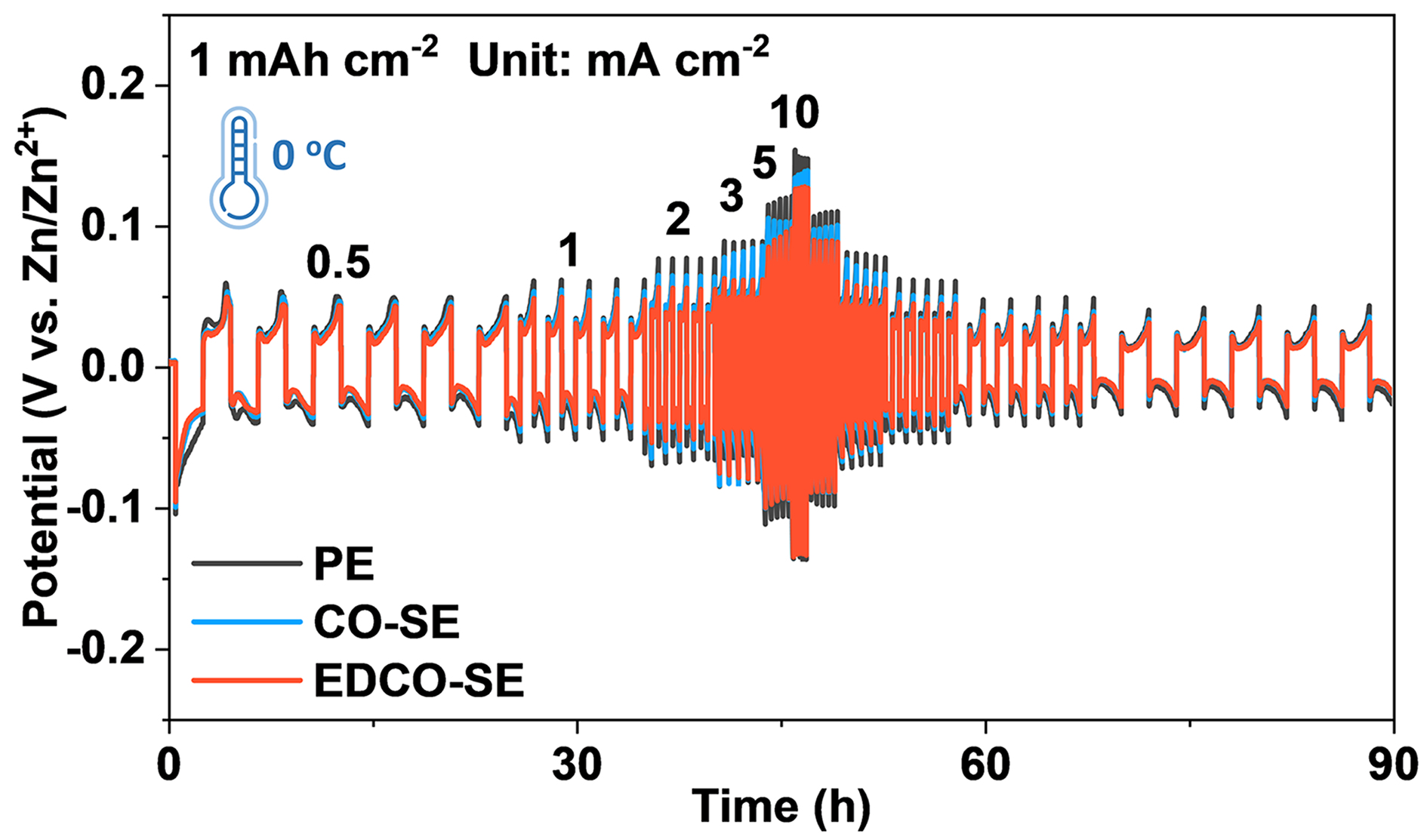


**Figure S26.** Rate performance from 0.5 to 10 mA cm^-2^ at 0 ^o^C.


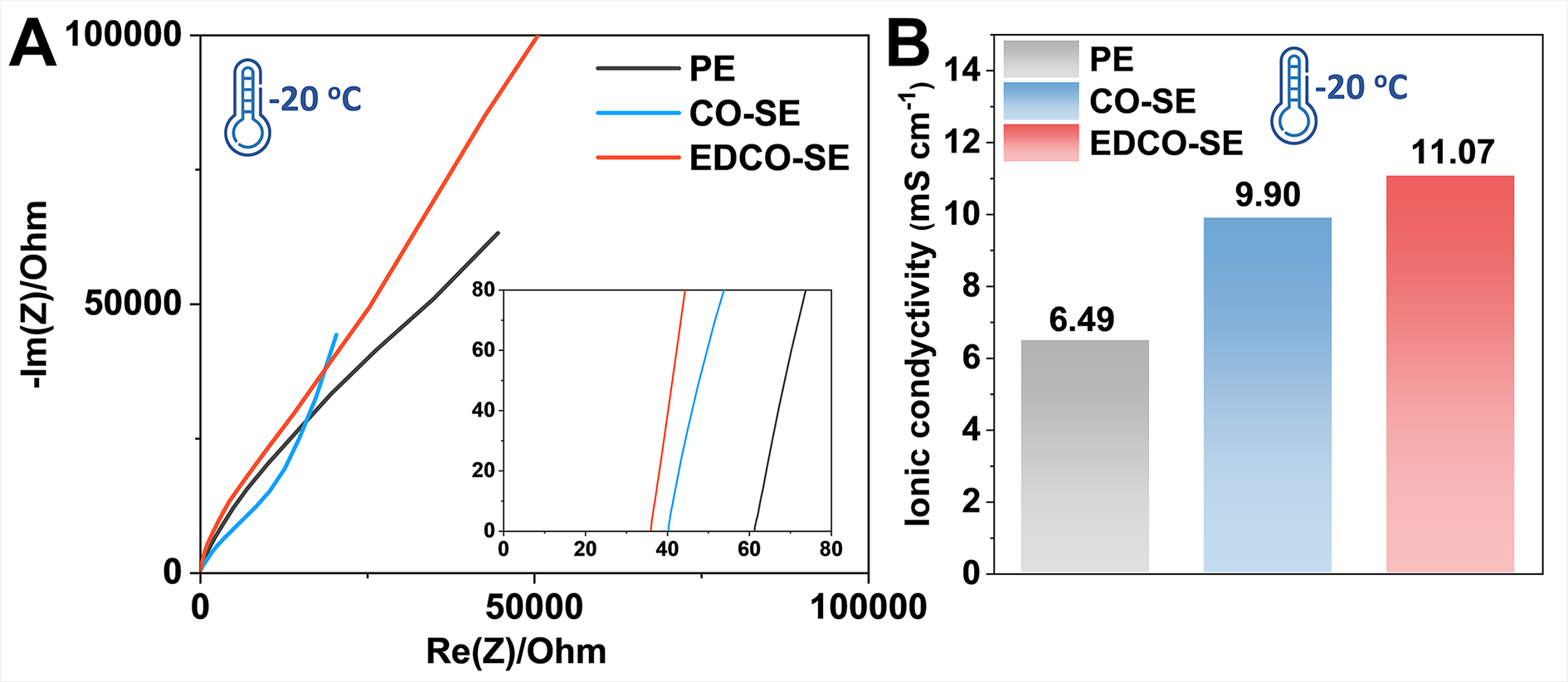


**Figure S27.** A) Ionic conductivity test of various electrolytes at -20 ^o^C. B) Ionic conductivity of various electrolytes at -20 ^o^C.


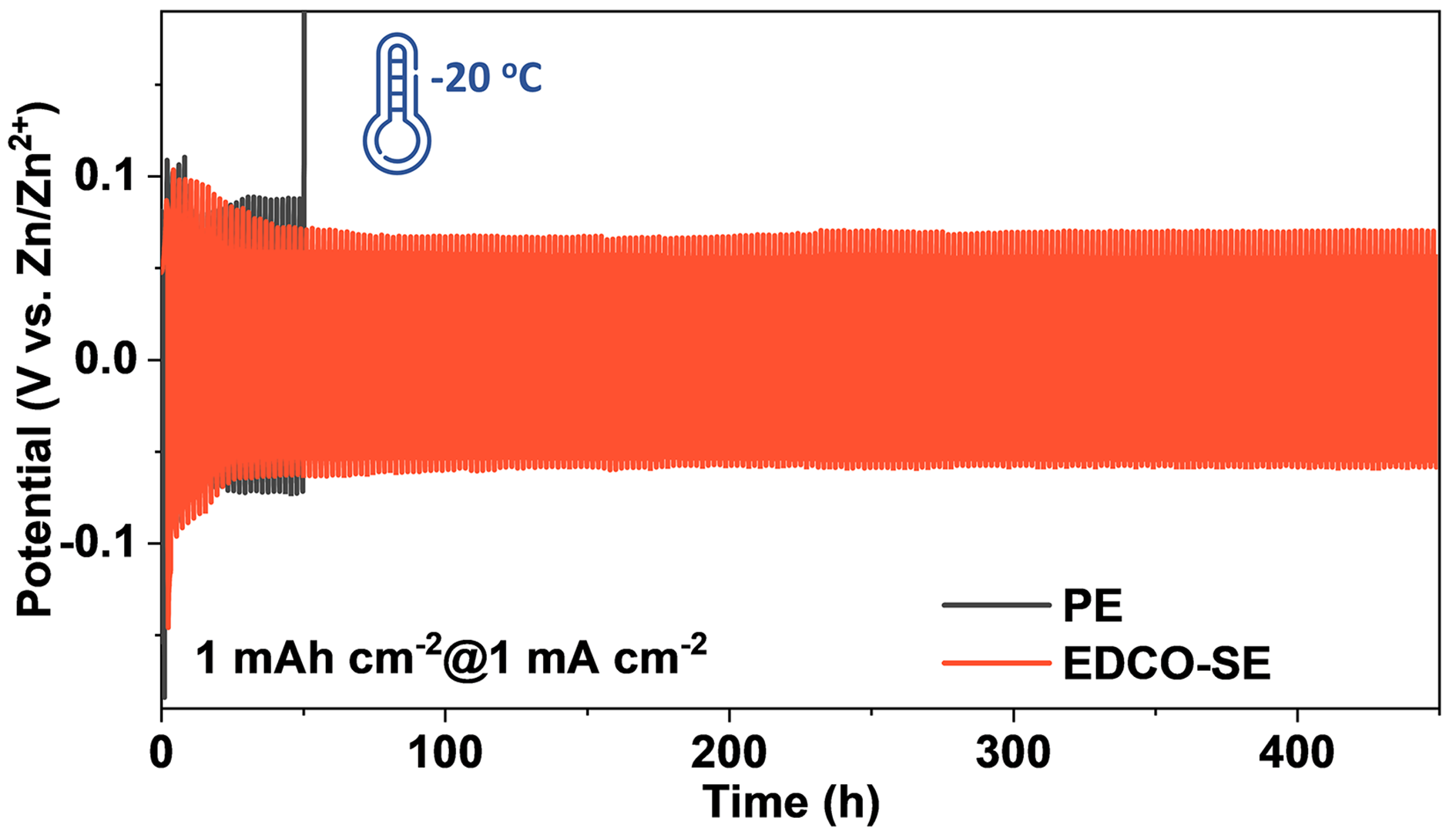


**Figure S28.** Long-term galvanostatic charge/discharge curves at -20 ^o^C.


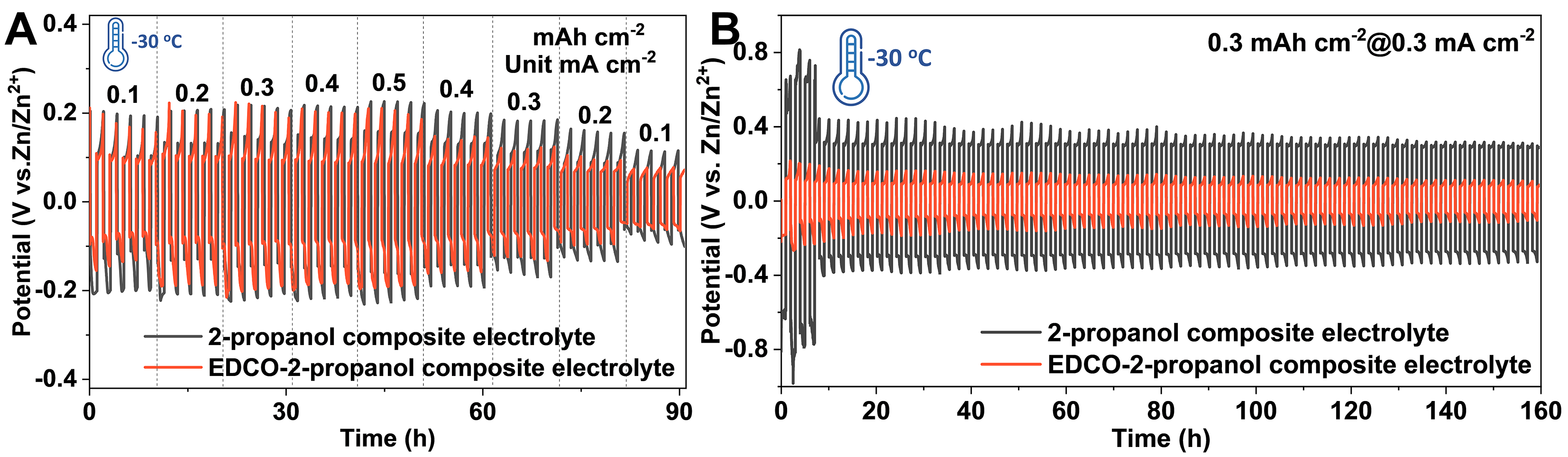


**Figure S29.** A) Rate performance and B) galvanostatic cycling of Zn symmetric cells at -30 ^o^C.


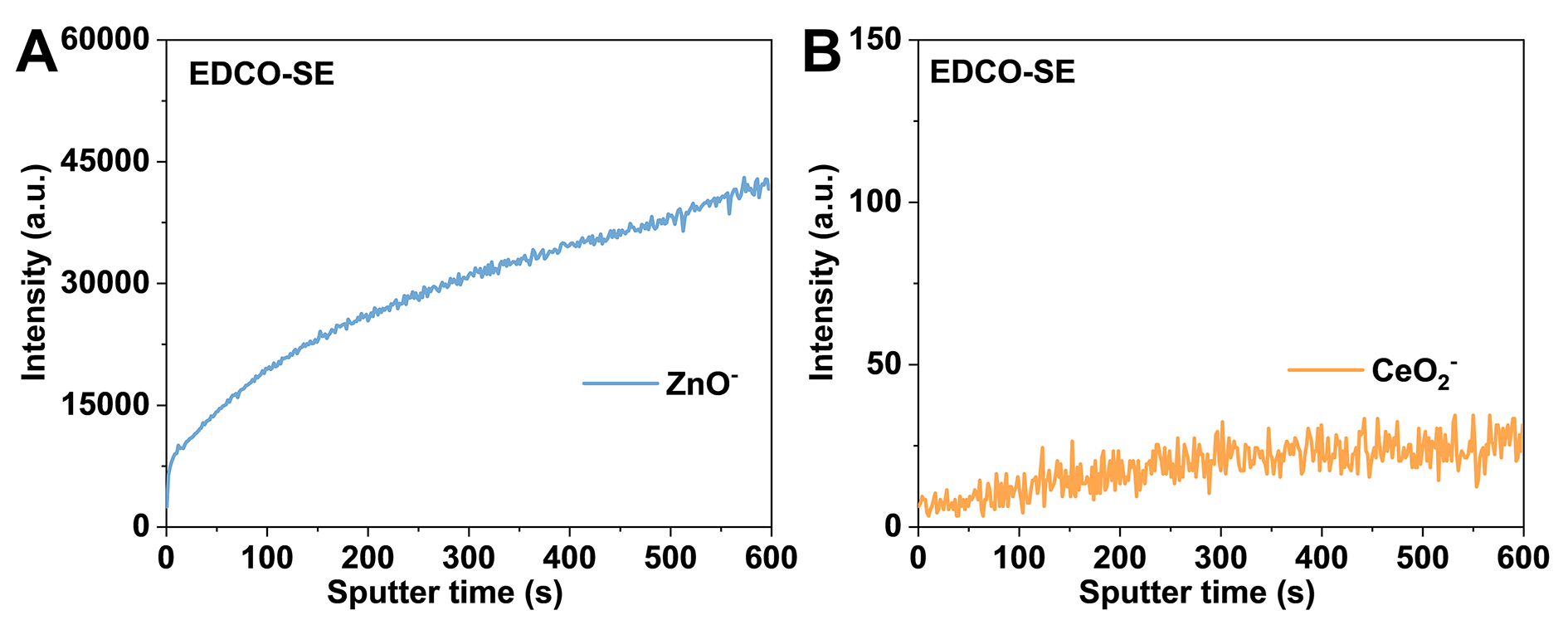


**Figure S30.** A) ZnO^-^ and B) CeO_2_^-^ depth profiles of the Zn anode in EDCO-SE as a function of etching time.





**Figure S31.** Cycling performance of full cells at 1 A g^-1^.





**Figure S32.** Cycling performance of full cells at 2 A g^-1^.





**Figure S33.** Rate performance of Zn||PANI full cells.


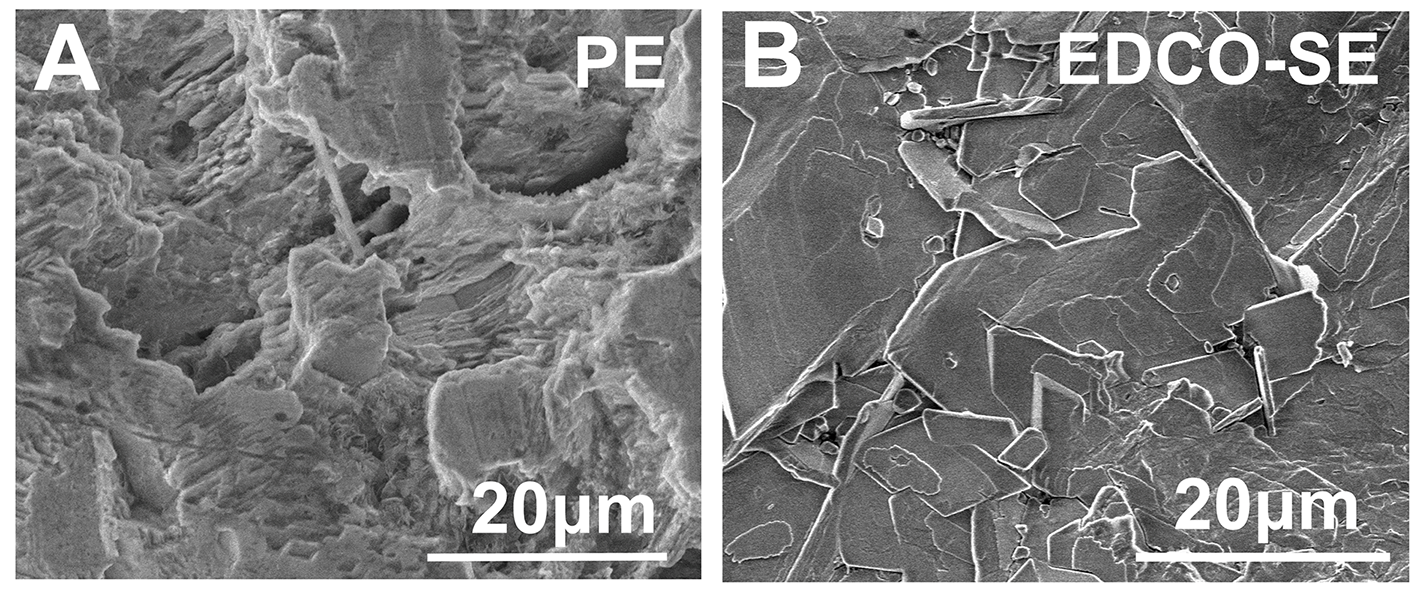


**Figure S34.** SEM images of the corresponded A) PE and B) EDCO-SE after 50 cycles.


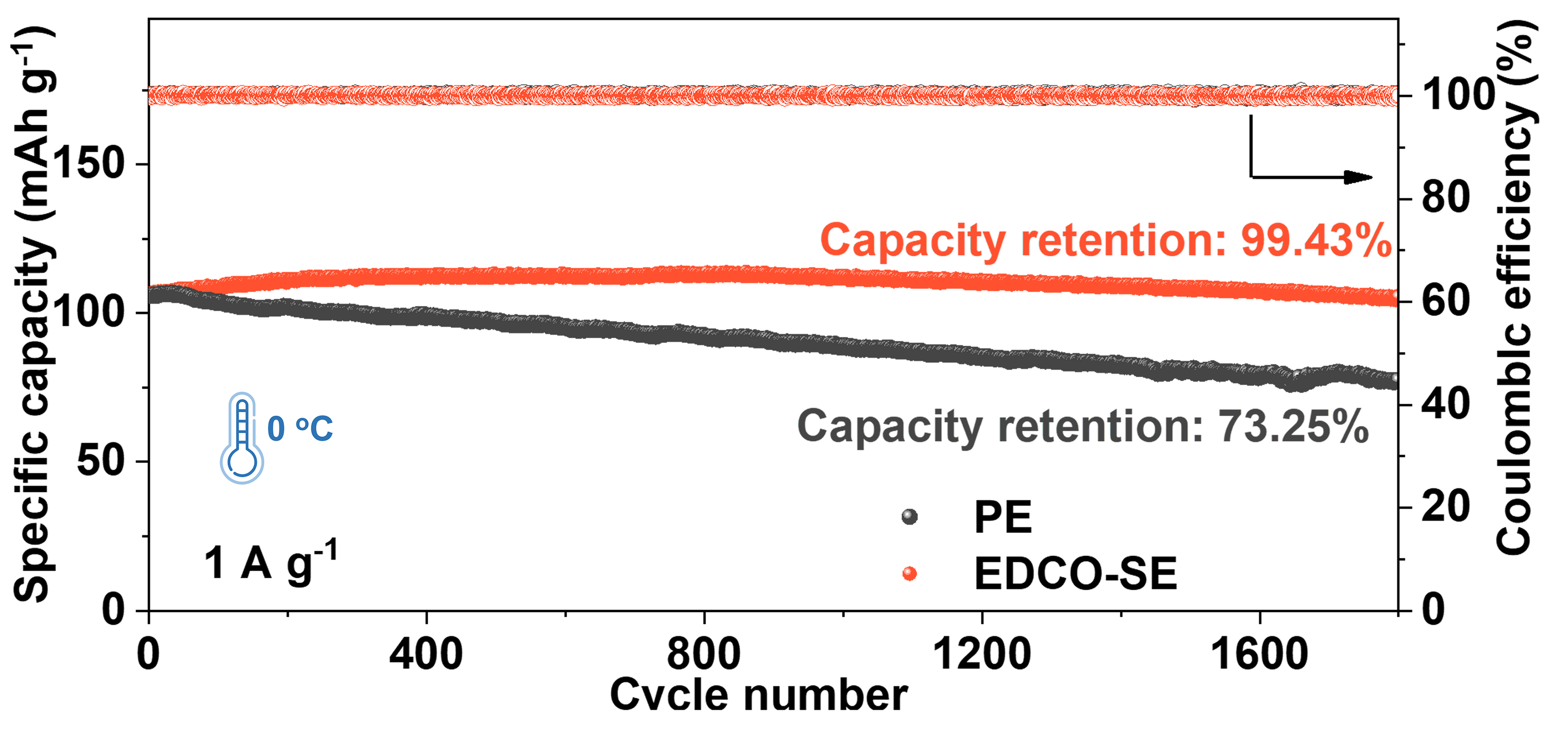


**Figure S35.** Cycling performance of Zn||PANI full cells at 0 ^o^C.


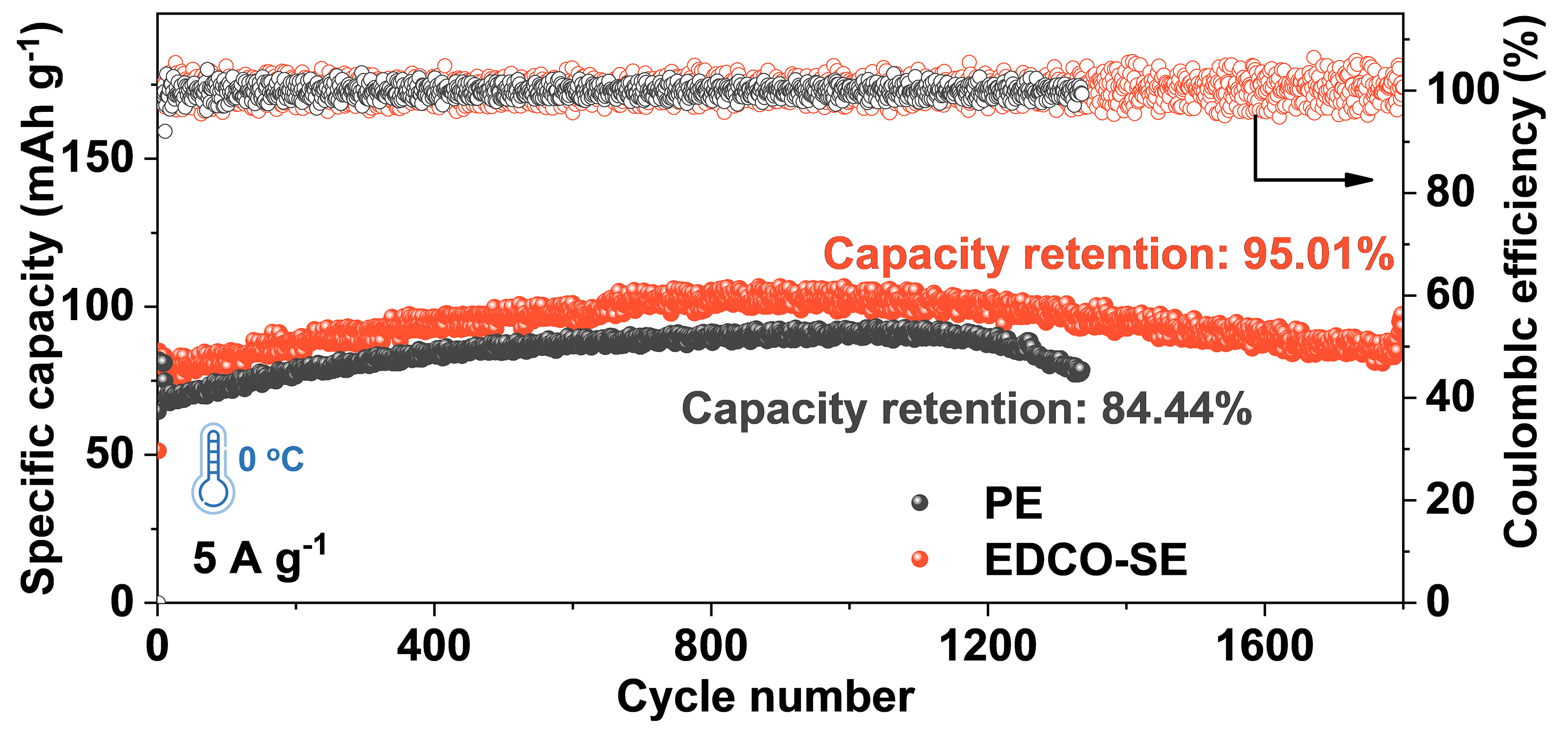


**Figure S36.** Cycling performance of full cells at 0 ^o^C.

**Table S1.** Cycle life and CE comparison with recently reported literatures utilizing different additives.

| **Electrolyte additive** | **Current density (mA cm^-2^)** | **Areal capacity (mAh cm^-2^)** | **Cycle number** | **CE (%)** | **Reference** |
| --- | --- | --- | --- | --- | --- |
| **EDCO** | **5** | **1** | **1200** | **99.92** | **Our work** |
| Glucose | 1 | 0.5 | 200 | 97.2 | *Angew. Chem.*, 2021, 133, 18395 |
| APA | 1 | 1 | 150 | 99.3 | *Energy Environ. Sci.*, 2023, 16, 1662 |
| TN-20 | 5 | 1 | 850 | 99.6 | *Adv. Funct. Mater.*, 2024, 34, 2311773 |
| q-GPA | 10 | 1 | 600 | 98.1 | *Adv. Energy Mater*., 2024, 2401470 |
| Propylene carbonate | 1 | 0.5 | 150 | 99.8 | *J. Am. Chem. Soc.*, 2022, 144, 7160 |
| Methylammonium acetate | 0.5 | 0.5 | 700 | 98.7 | *J. Comput. Chem.*, 1997, 18, 1463 |
| Na_4_EDTA | 0.5 | 0.5 | 300 | 98.4 | *Adv. Energy Mater.*, 2021, 11, 2102010 |
| Disodium lauryl phosphat | 1 | 1 | 550 | 98.2 | *Adv. Mater.*, 2024, 36, 2308577 |
| HMTA | 1 | 0.5 | 800 | 99.8 | *Adv. Energy Mater.*, 2023, 13, 2300550 |
| L-carnitine | 10 | 1 | 1000 | 98.1 | *Energy Environ. Sci.*, 2023, 16, 2684 |

**Table S2.** Performance of Zn symmetrical cells of our work and the reported works.

| **Electrolyte** | **Current density (mA cm^-2^)** | **Cycling**  **lifetime (h)** | **Reference** |
| --- | --- | --- | --- |
| **EDCO-SE** | **1** | **6500** | **Our work** |
| CMCS/PAM hydrogel | 1 | 1000 | *Adv. Mater.*, 2022, 34, e2110140 |
| C-ZnCl_2_/PG | 1 | 550 | *Energy Storage Mater.*, 2024, 70, 103524 |
| LA-Zn(OTf)_2_ | 1 | 800 | *Energy Storage Mater.*, 2024, 71, 103567 |
| Zn(BF_4_)_2_ | 0.5 | 1500 | *J. Mater. Chem., A.*, 2021, 9, 7042 |
| PAM ZS/GL/AN hydrogel | 0.2 | 500 | *Chem. Eng. J.*, 2022, 434, 134646 |
| ZnSO_4_+DMSO | 0.5 | 1200 | *Small*, 2021, 17, e2103195 |
| DLC/Sn-DLC@Zn (artificial layer) | 0.5 | 926 | *Adv. Energy Mater.*, 2024, 14, 2402015 |
| ZnF_2_-Ag@Zn (artificial layer) | 0.25 | 1600 | *Nano Lett.*, 2022, 22, 1750 |
| Zn@SIP (artificial layer) | 0.2 | 2100 | *Adv. Mater.*, 2022, 34, 2203153 |
| SZS@Zn (artificial layer) | 1 | 1000 | *Sci. Bull.*, 2025, 70, 518 |
| PVA@SR-ZnMoO_4_@Zn (artificial layer) | 2 | 2000 | *Energy Environ. Sci.*, 2023, 16, 275 |

**Table S3.** Cycle life and capacity retention are compared with recently reported literature using different additives.

| **Electrolyte** | **Cathode** | **Current density** | **Cycle number** | **Capacity retention (%)** | **Reference** |
| --- | --- | --- | --- | --- | --- |
| **EDCO-SE** | **PANI** | **1 A g^-1^** | **2300** | **86.74** | **Our work** |
|  |  | **2 A g^-1^** | **2800** | **84.21** |  |
| (Zn(OAc)_2_)-FA | PANI | 1 A g^-1^ | 800 | 60 | *Energy Environ. Sci.*, 2023, 16, 5096-5107 |
| Methanol-50% | PANI | 5 A g^-1^ | 2000 | 85.5 | *Angew. Chem. Int. Ed.*, 2021, 60, 7366-7375 |
| Zn(OTf)_2_-γ-valerolactone | PANI | 2 mA cm^-2^ | 1600 | 85 | *Sci. Bull.*, 2023, 68,1531-1539 |
| Zn(BF_4_)_2_-AN | PANI | 1 A g^-1^ | 2000 | 86.72 | *J. Am. Chem. Soc.*, 2024, 146, 31612-31623 |
| ZnSO_4_-EMImCl | Z-PANI | 1 A g^-1^ | 300 | 78.8 | *Angew. Chem.*, 2021, 133, 23545-23552 |
| ZnSO_4_-EG | PANI-V_2_O_5_ | 1 A g^-1^ | 300 | ~51 | *Energy Environ. Sci.*, 2020, 13, 3527-3535 |
| Zn(CF_3_SO_3_)_2_ | MEG/PANI | 2 A g^-1^ | 2000 | 72.7 | *Chem. Eng. J.*, 2022, 440, 135930 |
